# Bistability of Dielectrically Anisotropic Nematic Crystals and the Adaptation of Endothelial Collectives to Stress Fields

**DOI:** 10.1002/advs.202102148

**Published:** 2022-03-28

**Authors:** Georgios Stefopoulos, Tobias Lendenmann, Thomas M. Schutzius, Costanza Giampietro, Tamal Roy, Nafsika Chala, Fabio Giavazzi, Roberto Cerbino, Dimos Poulikakos, Aldo Ferrari

**Affiliations:** ^1^ Laboratory of Thermodynamics in Emerging Technologies, Department of Mechanical and Process Engineering ETH Zurich Sonneggstrasse 3 Zurich 8092 Switzerland; ^2^ Experimental Continuum Mechanics EMPA, Swiss Federal Laboratories for Materials Science and Technology Überlandstrasse 129 Dübendorf 8600 Switzerland; ^3^ Institute for Mechanical Systems, Department of Mechanical and Process Engineering ETH Zurich Leonhardstrasse 21 Zurich 8092 Switzerland; ^4^ Dipartimento di Biotecnologie Mediche e Medicina Traslazionale Università degli Studi di Milano Via F.lli Cervi 93 Segrate 20090 Italy; ^5^ Faculty of Physics University of Vienna Boltzmanngasse 5 Vienna Austria

**Keywords:** collective cell behavior, endothelia, monolayers, nematic, polarization, wall shear stress

## Abstract

Endothelial monolayers physiologically adapt to flow and flow‐induced wall shear stress, attaining ordered configurations in which elongation, orientation, and polarization are coherently organized over many cells. Here, with the flow direction unchanged, a peculiar bi‐stable (along the flow direction or perpendicular to it) cell alignment is observed, emerging as a function of the flow intensity alone, while cell polarization is purely instructed by flow directionality. Driven by the experimental findings, the parallelism between endothelia is delineated under a flow field and the transition of dual‐frequency nematic liquid crystals under an external oscillatory electric field. The resulting physical model reproduces the two stable configurations and the energy landscape of the corresponding system transitions. In addition, it reveals the existence of a disordered, metastable state emerging upon system perturbation. This intermediate state, experimentally demonstrated in endothelial monolayers, is shown to expose the cellular system to a weakening of cell‐to‐cell junctions to the detriment of the monolayer integrity. The flow‐adaptation of monolayers composed of healthy and senescent endothelia is successfully predicted by the model with adjustable nematic parameters. These results may help to understand the maladaptive response of in vivo endothelial tissues to disturbed hemodynamics and the progressive functional decay of senescent endothelia.

## Graphical abstract

The collective organization of endothelial cells is instructed by the flow direction and intensity, a critical aspect of the cell assembly functionality. The dynamic variation of the external flow field exposes the cellular system to a disruptive level of disorder. The emerging new picture defines endothelia as unique active tissues with their own assembly identity, characterized by an architectural hysteresis.

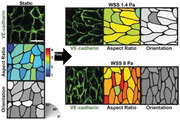

## 1 Introduction

Understanding how cells interact with one another and with their external environment is a necessary, fundamental step in our quest to predict and influence the collective fate of multicellular assemblies such as embryos, tissues, and organs.^[^ ^1^, ^2^ ^]^ Simple models and analogies stemming from inert soft matter physics have been successfully adapted to describe such interactions and to formulate testable predictions relying on key control parameters.^[^ ^3^, ^4^, ^5^, ^6^ ^]^

A successful example of this adaptation is represented by the description of confluent epithelial cell monolayers as soft materials in which a combination of intrinsic anisotropy and self‐propulsion generates local liquid crystalline order, similar to what occurs in a dense collection of rod‐shaped molecules.^[^ ^5^ ^]^As in inert liquid crystals, the nematic order of epithelia can be locally frustrated yielding topological defects that coincide with the extrusion of cells from the monolayer.^[^ ^7^ ^]^ The presence of nematic alignment and topological defects was also observed in other cell types including fibroblasts.^[^ ^8^, ^9^ ^]^ In this system, analogies with the Fréedericksz transition of liquid crystals were useful to explain spontaneous shear flows arising in confined monolayers and their dependence on the confinement width.^[^ ^10^ ^]^

Much less investigated is the usefulness of soft‐matter inspired physical models in describing the behavior of endothelia, a dense cell system actively responding to flow.^[^ ^11^ ^]^ Endothelial cells, like epithelial ones, grow to confluence and mature forming a mechanically and biologically‐connected monolayer.^[^ ^12^ ^]^ In vivo, endothelia occupy the interface between the lumen of blood and lymphatic vessels and the surrounding tissues.^[^ ^13^ ^]^ They are naturally exposed to unidirectional flow and flow‐generated wall shear stress (WSS). The local hemodynamic provides anisotropic signals fundamentally contributing to tissue maturation and function.^[^ ^14^ ^]^

Flow induces coordinated planar cell polarity (PCP), whereby cells coherently re‐localize cellular compartments, such as the Golgi, and functions.^[^ ^15^ ^]^ In addition, they adaptively elongate and orient the cell body.^[^ ^16^ ^]^ Endothelia exposed to unidirectional flow in vitro, polarize to the upstream direction (i.e., against the flow).^[^ ^15^ ^]^ Parallel cell elongation is typically observed under low WSS values.^[^ ^17^ ^]^ At higher WSS levels, collective cell arrangement falls in a different regime, and perpendicular (to flow) orientation emerges.^[^ ^18^, ^19^ ^]^ These two orthogonal orientations are similarly observed in vivo. The specific angle of alignment and the direction of PCP vary depending on the vascular bed, age, and local hemodynamic conditions,^[^ ^20^ ^]^ with perpendicular alignment observed in locations of the body exposed to high flow, as on the surface of heart valves.^[^ ^21^ ^]^ Disturbed hemodynamics can compromise this coherent multicellular adaptation.^[^ ^11^ ^]^ In vivo, regions of vessel stenosis or bifurcation, prone to flow recirculation typically feature cells with random PCP and orientation. These locations represent hotspots for the development of inflammatory processes leading to a progressive functional decay typical of endothelial senescence.^[^ ^22^, ^23^ ^]^ Senescent endothelial cells accumulate genetic, metabolic, and structural damage and lose the ability to respond to flow.^[^ ^24^ ^]^ Regions of the vasculature affected by senescence are exposed to endothelial denudation and atherosclerosis.^[^ ^20^ ^]^

Here, to describe the complex endothelial response to flow, we employed a custom‐developed bioreactor^[^ ^19^ ^]^ and exposed human cells to laminar flow generating low (1.4 Pa) or high (8 Pa) WSS. We monitored two alternative transitions yielding orthogonal collective cell alignment (bistability) but identical PCP. In both cases, the achievement of coherent organization required dynamic force generation and a transient increase of junctional tension in the monolayer.^[^ ^25^ ^]^

To explore the physics of this complex behavior, we propose a simple analogy with a dielectrically anisotropic nematic liquid crystal under the action of an oscillating external electric field. The model features a collection of polar anisotropic elements—whose polarity along the long and short axis depends on the frequency of the applied electric field, termed hereafter as a dual‐frequency liquid crystal—and incorporates the key symmetries of the problem. Adaptation of the system to an external oscillatory electric field with variable frequency generates two distinct ordered phases characterized by identical polarization but orthogonal alignment of the elements. The model allows one to simulate the transition between these two ordered phases, revealing the existence of an intermediate, disordered state slowly relaxing to an ordered configuration. The emergence of such transitional arrangement in endothelial monolayers was experimentally verified: a sudden switch in the WSS level induced a randomization of both ordered states, which evolved into an isotropic configuration where collective alignment was lost. This development was rapidly followed by the disassembly of cell‐to‐cell junctions and loss of monolayer integrity. Based on the analogy with dual‐frequency nematics, the transition to an intermediate disordered state can be explained by the inability of cells to simultaneously satisfy requirements of shape and orientation on the one hand, and polarity with respect to the external field, on the other hand, within the time frame in which the monolayer maintains its integrity. The resulting physical picture has its origin in the active nature of cells and in the intrinsic functional polarity of endothelia, piecing together a generic mechanism to interpret the effects of hemodynamic perturbations on human tissues.

## 2 Results

**Figure 1:**
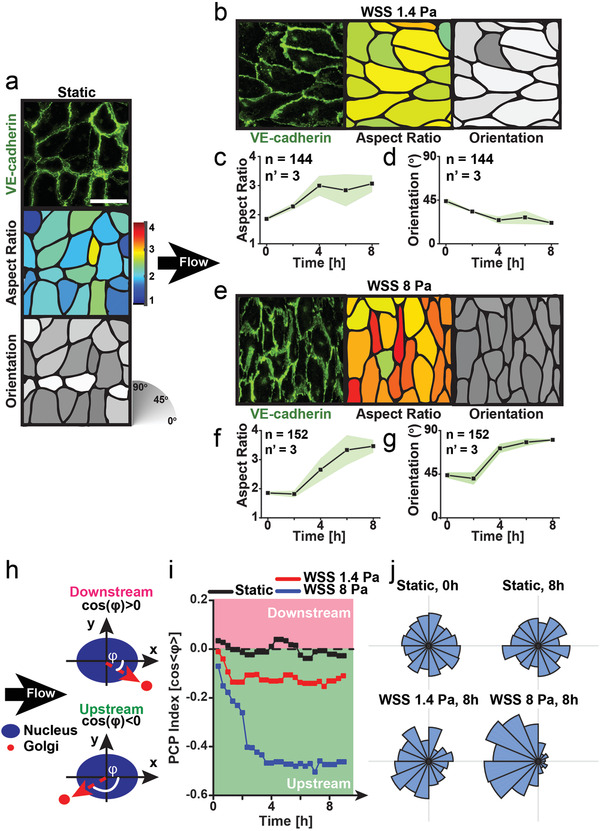
Alternative transitions in endothelial monolayers under flow. a) Isotropic state in static conditions and b–g) adaptation to unidirectional flow generating low (i.e., 1.4 Pa) or high (i.e., 8 Pa) WSS. Fluorescent images of VE‐cadherin distribution (green) at cell‐to‐cell junctions (top panel in (a), leftmost panel in (b) and (e)). Scale bar is 50 µm. Cell profiles with color‐coded aspect ratio (middle panel in (a), (b), and (e)) and cell orientation (bottom panel in (a) and rightmost panel in (b) and (e)) as encoded by corresponding color scale bars. (b) Anisotropic state featuring cell alignment along the direction of flow. Corresponding evolution of cell aspect ratio (c) and orientation. (e) Anisotropic state featuring cell alignment orthogonal to flow. Evolution of cell aspect ratio (f) and orientation (g). In isotropic system configurations cells tend to be round (i.e., lower aspect ratio) and randomly oriented (i.e., at 45°). The number of analyzed cells is reported as n and the number of independent experiments as n’. The green shaded area indicates the standard deviation. h) Cartoon defining PCP under flow. i) Evolution of PCP in static conditions and upon exposure to unidirectional flow generating WSS values of 1.4 or 8 Pa. j) Radial distribution of PCP (i.e., *ϕ* as defined in (h)) in static conditions at 0 (top left panel) and 8 h (top right panel) and under flow generating WSS of 1.4 (bottom left panel) or 8 Pa (bottom right panel), both at 8 h.

To investigate the self‐organization ability of human endothelia we monitored their response to flow (**Figure** **1**). Monolayers developed in static culture featured cells with weak shape anisotropy and random PCP (Figure S1 and Video S1, Supporting Information), a condition that represented the starting point for the flow experiments (Figure 1a). A fully developed laminar flow coupled to the monolayers of interest was generated in an optically conducive bioreactor.^[^ ^19^ ^]^ Endothelia were exposed to WSS of 1.4 or 8 Pa. Onset of flow prompted a fast response (Videos S2 and S3, Supporting Information), which induced cell shape elongation and collective orientation (Figure 1b–g). During the adaptation to flow, PCP was visualized by the position of Golgi relative to the nucleus.^[^ ^15^ ^]^ Coherent cell polarity was accomplished within ≈2–4 h (Figure 1h–j). Cell shape change and collective alignment proceeded with slower dynamics reaching a plateau after ≈6–8 h (Figure 1b–g). At this point, cell ensembles featured a statistically stable collective organization that was maintained for extended exposures to unaltered flow.

For low WSS values (i.e., 1.4 Pa) cell bodies elongated along the flow (parallel alignment; Figure 1b–d, Figure S12, Supporting Information). Exposure to high WSS (i.e., 8 Pa), prompted instead a faster remodeling yielding perpendicular cell alignment (≈90° to the direction of flow; Figure 1e–g, Figure S12, Supporting Information). Interestingly, despite orthogonal orientation, both ordered states featured identical PCP with Golgi positioned upstream of the nucleus (upstream polarity; Figure 1i,j and Figure S2, Supporting Information). These emerging responses were not observed in isolated endothelial cells exposed to the same hydrodynamic conditions (Video S4, Supporting Information).

Taken together, these results confirm that endothelial cells in confluent monolayers coordinately adapt shape, alignment, and polarity in response to flow. Collective cell body elongation and alignment depends on the WSS level. On the other hand, PCP reflects flow directionality (Figure 1). To assess whether cell orientation and polarity represent independent collective properties of mature endothelia, we generated monolayers on anisotropic substrates in static conditions.^[^ ^19^ ^]^ We exploited micro‐engineered substrates featuring a surface geometry in the form of gratings. Topographic contact guidance was effective in inducing cell elongation and alignment along the direction of the gratings.^[^ ^26^ ^]^ The collective cell alignment was equivalent to the one induced by flow,^[^ ^19^ ^]^ but was obtained in static conditions. Therefore, notwithstanding the strong and coherent orientation of cell shape, PCP remained randomly distributed (Figure S2, Supporting Information) demonstrating that the two properties can be fully decoupled. This demonstrates that cell alignment and PCP are independent and can be acquired by cells in response to different external stimuli, or combinations of them.

**Figure 2:**
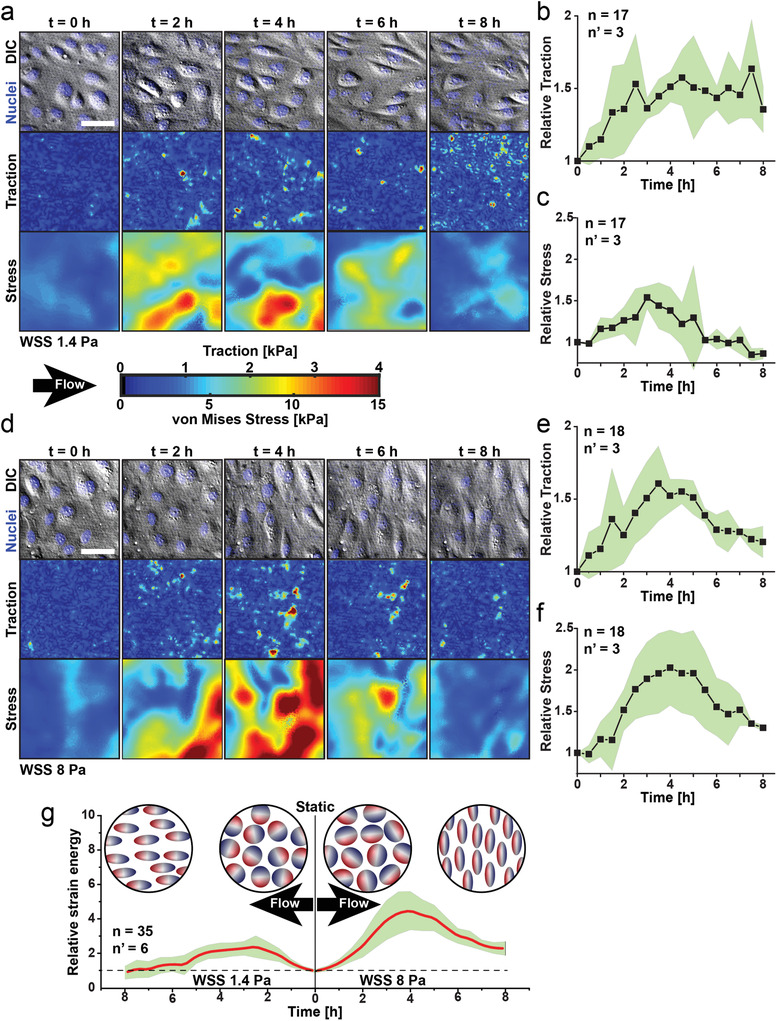
Traction and stress during alternative transitions in endothelial monolayers under flow. a) DIC images and fluorescent nuclei (top row), traction force (middle row) and intercellular stress (bottom row) maps for endothelia exposed to unidirectional flow generating WSS of 1.4 Pa. Scale bar is 50 µm. Relative evolution of b) traction force and c) intercellular stress. d) Corresponding DIC and nuclei (top row) and traction and stress maps (middle and bottom rows; respectively) for endothelial exposed to unidirectional flow generating WSS of 8 Pa. The flow direction is indicated by a black arrow. The magnitude of traction and intercellular stress is indicated by the color scale bar. Relative evolution of e) traction and f) stress. Mean values (black squares) are plotted (b,c,e,f). g) Relative strain energy produced by the endothelium during the transition from the initial isotropic state (static) to the alternative anisotropic states. The shaded green area corresponds to the standard deviation. A corresponding representation of the endothelial monolayer states is displayed in the circular cartoons. The number of analyzed fields is reported as n and the number of independent experiments as n’.

We next investigated the dynamics of monolayer transition to either of the two flow‐driven ordered states (**Figure** **2**). Confocal traction force microscopy (cTFM), a recently introduced method to obtain a direct visualization of cell tractions on compliant substrates,^[^ ^27^, ^28^, ^29^ ^]^ was employed. Cells transmitted distinctive patterns of traction to the substrate during collective orientation and polarization (Figure 2a,d). A force peak was measured between 2 and 6 h from the onset of flow (Figure 2a,b,d,e). The system relaxed as soon as the majority of cells reached the final orientation, shape, and PCP. In addition, using monolayer stress microscopy,^[^ ^30^ ^]^ intercellular stresses were mapped (Figure 2c,f), further revealing tension maxima during the adaptation phase. Traction and stress peaks were significantly higher during evolution to perpendicular alignment (WSS = 8 Pa), yet the trend and dynamics were similar between the two orthogonal transitions. Intercellular stresses similarly relaxed upon attainment of global order.^[^ ^31^ ^]^

Liquid crystal models have proven helpful in describing phase behavior and transitions in epithelial monolayers, as a function of cell density, anisotropy, and self‐propulsion.^[^ ^5^, ^7^, ^10^, ^32^ ^]^ Such models, however, do not include an intrinsic polarity and can therefore not distinguish between states with orthogonal collective orientation. On the other hand, models of liquid crystals formed by polar elements^[^ ^33^ ^]^ contemplate alternative transitions of anisotropic particles with positive or negative dielectric anisotropy Δ*ε*, when exposed to an external electric field. This transition is triggered by the frequency of an oscillatory electric field for a dual‐frequency liquid crystal. An early model of the dielectric relaxation was provided by Debye^[^ ^34^ ^]^ where the dielectric constant *ε* varies with *ω* according to

(1) $$\varepsilon =\varepsilon_{\infty }+\frac{\varepsilon_{0}-\varepsilon_{\infty }}{1+{\left(\frac{\varepsilon_{0}+2}{\varepsilon_{\infty }+2}\omega \tau \right)}^{2}},$$

and where *τ* is the dipole relaxation time. Subscripts “0” and “∞” indicate the values corresponding to *ω* = 0 and *ω*→∞, respectively. We used a somewhat simpler but essentially similar model for the *ω*‐dependence of *ε*_∥_, the dielectric constant of the dual‐frequency liquid crystal molecules along the long molecular axis, given by^[^ ^35^ ^]^

(2) $$\varepsilon_{∥}=\varepsilon_{∥,\infty }+\frac{\varepsilon_{∥,0}-\varepsilon_{∥,\infty }}{1+{\left(\omega \tau \right)}^{2}},$$

The dielectric constant along the short molecular axis *ε*_⊥_ is independent of *ω*. Therefore, the dielectric anisotropy Δ*ε* = *ε*_∥_ − *ε*_⊥_ changes sign from positive (corresponding to *ω* < *ω*_0_) to negative (corresponding to *ω* > *ω*_0_) with a zero‐crossing (at *ω* = *ω*_0_). We adapted this model of the frequency‐dependent dipole moment in order to describe the phase‐transition of the endothelial monolayer. We used *ε*_∥,0_ = 0.5, *ε*_∥,∞_ = 0.01, *ε*_⊥_ = 0.2, and *τ* = 0.6 *s*, which ensures a positive dielectric anisotropy (Δ*ε* = 0.16) at low‐*ω* (*ω*_*L*_ = 1.1 *s* ^−1^, corresponding to WSS = 1.4 Pa), a negative dielectric anisotropy (Δ*ε* = − 0.15) at high‐*ω* (*ω*_*H*_ = 6.3 *s* ^−1^, corresponding to WSS = 8 Pa) with a zero‐crossing at *ω*_0_ = 2.1 *s* ^−1^. The numerical values of the parameters are chosen to ensure a Fréedericksz transition ($\frac{d^{2}}{\pi^{2}}\frac{\Delta \varepsilon }{R_{1}}\geq \frac{1}{E^{2}}$, where *d* is the domain size and *R*_1_ is the elastic modulus)^[^ ^36^ ^]^ and focusing mainly on the demonstration of the phase‐transition of the dual‐frequency liquid crystal system.

**Figure 3:**
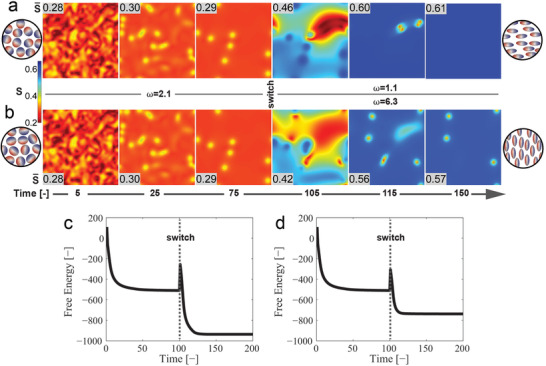
Numerical simulation of system transitions in dual‐frequency nematic liquid crystals exposed to an oscillatory electric field with frequency *ω*. a) Heat maps of the transient nematic order parameter, *S*, for switching of *ω* from *ω_0_* to *ω_L_* (Δ*ε* > 0; the mean value $\bar{S}$ is inset). b) Corresponding heat maps for switching of *ω* from *ω_0_* to *ω_H_* (Δ*ε* < 0). Both (a) and (b) are initiated with the same random configuration. At time = 100 the frequency change is triggered (switch). A corresponding representation of the initial (left) and final (right) states is displayed in the circular cartoons. The magnitude of *S* is indicated by the color scale bar. c) Free energy profile during the system transition depicted in (a). d) Corresponding free energy profile during the transition depicted in (b).

A 2D liquid crystal formed by weakly nematic elements was initially allowed to equilibrate in silico to its ground state with *E_x_* = 0 and *ω* = *ω*_0_ (**Figure** **3**a,b). During this phase, the nematic order parameter increased as a function of time (from time = 0 to 100, where the kinetic time constant Γ = 1), enabling the attainment of nematic order, akin to what is described for epithelial monolayers.^[^ ^8^ ^]^ The simulations presented here are representative outcomes, and the reported qualitative behavior was found to be robust for 0 ≤ *ω* < ∞, 0.3 ≤ *E_x_* ≤ 1.5 and 0.02 ≤ |Δ*ε*| ≤ 0.36 (Figures S6–S10, Supporting Information). The switch of *ω* (and thereby the sign and magnitude of Δ*ε*) triggered a global configuration change, with a sharp increase of disorder (time ≈ 100) followed by a monotonous relaxation to a highly ordered state (time > 100). Specifically, the nematic director aligned along the field direction if *ω* = *ω*_*L*_ < *ω*_0_, while orthogonal alignment was obtained when *ω* = *ω*_*H*_ > *ω*_0_. Both ordered states featured identical polarization. (See Figure S11, Supporting Information, Section S2, Supporting Information, for a description of the analogy between the in silico model and in vitro experiments.)

The qualitative agreement with the reported response of endothelial monolayers exposed to flow (Figure 1) was further investigated and validated by the direct measurement of the energy landscape characterizing the alternative transitions. Using the Landau‐de Gennes theory and adapting a previously published, open‐source algorithm,^[^ ^33^ ^]^ we modeled the relaxation dynamics of dual‐frequency liquid crystals under applied electric fields and the corresponding change in free energy of the system with time (see Supplementary Information, section “Modeling Nematic Liquid Crystals”). For both transitions (Figure 3a,b), a free energy peak, indicative of a non‐equilibrium state, was reached upon the frequency switch and was relaxed (decreased) as soon as a high level of order was obtained (Figure 3c,d). These trends were compared with the relative strain energy measured upon endothelial adaptation to flow, yielding a similar bimodal energy profile, characterized by an initial increase followed by relaxation (Figure 2c,f and Figure 3c,d). This analogy suggests that the transient dynamics exhibited by a dielectrically anisotropic nematic liquid crystal responding to the frequency of an external oscillatory field may correspond to the observed peaks of junctional tension in endothelia adapting to flow (Figure 2c,f).

**Figure 4:**
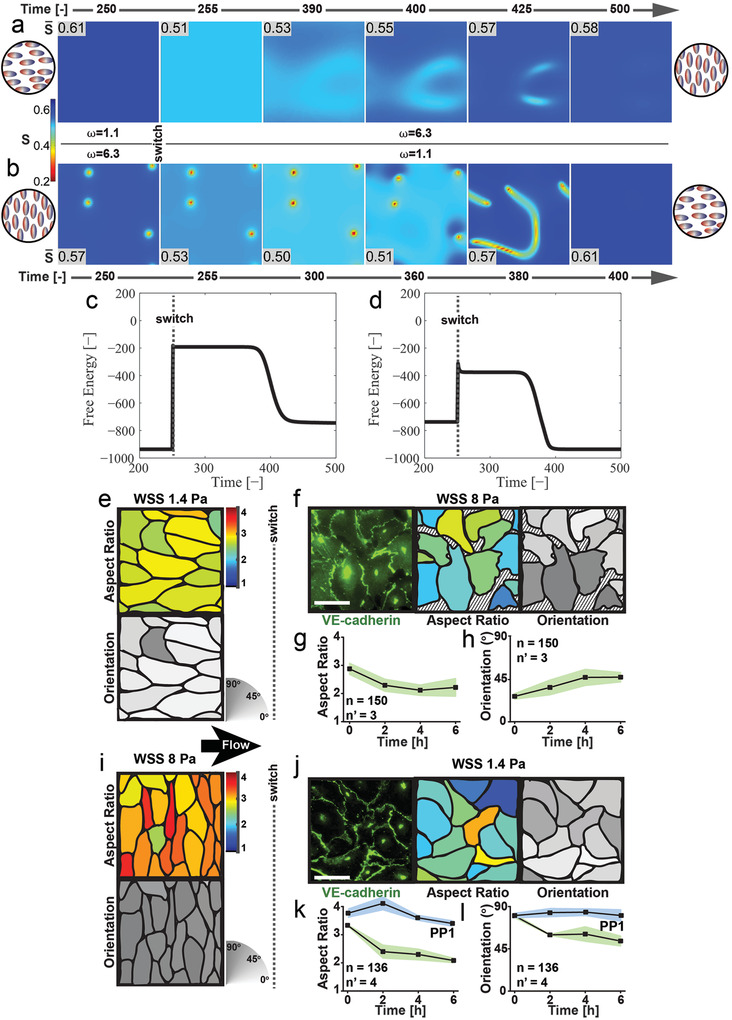
System transitions upon perturbation (a–d: in silico, e‐l: in vitro). a) Heat maps of the transient nematic order parameter, *S*, for polar nematic crystal in the presence of an oscillatory electric field upon switching frequency from *ω*_*L*_ to *ω*_*H*_ (the mean values, $\bar{S},$ are inset). b) Corresponding heat maps of *S* for the opposite switch, that is, from *ω*_*H*_ to *ω*_*L*_. The switch occurs at time = 250. A corresponding representation of the initial (left) and final (right) states is displayed in the circular cartoons. c) Free energy development during the system transition depicted in (a). d) Corresponding free energy development during the transition depicted in (b). e) Anisotropic state featuring cell alignment along the direction of flow generating physiological (i.e., 1.4 Pa) WSS and f–h) rearrangement in response to a WSS increase (from 1.4 to 8 Pa). Fluorescent images of VE‐Cadherin distribution (green) at cell‐to‐cell junctions (top panel in (a) and (i), leftmost panel in (f) and (j)). Scale bar is 50 µm. Cell profiles with color‐coded aspect ratio (middle panel in (e), (f), and (j)) and cell orientation (bottom panel in (e) and (i) and rightmost panel in (f) and (j)) as encoded by corresponding color scale bars. (f and j) Isotropic intermediate states featuring intercellular gaps (regions identified by black stripes). Corresponding evolution of cell aspect ratio (g and k) and orientation (h and l). In isotropic system configurations cells tend to be round (i.e., lower aspect ratio) and randomly oriented (i.e., at 45°). i) Anisotropic state featuring cell alignment perpendicular to the direction of flow generating supraphysiological (i.e., 8 Pa) WSS and j–l) rearrangement in response to WSS decrease (from 8 to 1.4 Pa) without and with (PP1) Src inhibition. The number of analyzed cells is reported as n and the number of independent experiments as n’. The shaded area indicates the standard deviation.

We next used the model to study the evolution of either ordered state when forced to develop into the orthogonal configuration (**Figure** **4**). This transition was induced by switching *ω* (at time = 250), from *ω*_*L*_to *ω*_*H*_ (Figure 4a) or from *ω*_*H*_ to *ω*_*L*_ (Figure 4b). In both cases, the system rapidly evolved to a configuration with higher disorder, from which it slowly escaped, relaxing into the alternative ordered state. The energy profile of these evolutions indicates the transition into a relatively persistent, metastable state characterized by a high level of free energy (Figure 4c,d).

The same transitions were experimentally reproduced in endothelial monolayers. Perturbation of fully aligned endothelia (Figure 4e,i) was obtained by sharply increasing (Figure 4e–h) or decreasing (Figure 4i–l) the WSS. The multicellular systems quickly reacted evolving towards an isotropic state where coherent cell alignment and elongation were lost (Figure 4f–h,j–l). Cell polarity was not significantly affected by the perturbation (Figure S3, Supporting Information). To assess whether the increase in disorder affected the junctional stability in the monolayer, the strength of cell‐to‐cell junctions during the transition was evaluated through the immunostaining of VE‐cadherin distribution and phosphorylation.^[^ ^14^ ^]^ This analysis revealed that all tested hydrodynamic perturbations significantly increased junctional VE‐cadherin phosphorylation and thus decreased the stability of junctions (Figure S4, Supporting Information). The transition to the isotropic intermediate state was, in fact, non‐conducive to further development and led to a loss of monolayer integrity, with the formation of gaps between cells (Figure 4) and ensuing cell loss (Video S5, Supporting Information). When Src tyrosine kinase, the enzymatic activity responsible for VE‐cadherin phosphorylation,^[^ ^14^ ^]^ was inhibited, the system transition upon WSS switch was totally repressed (Figure 4k,l). In addition, when the WSS switch was completed by gradually changing the flow intensity, the intermediate isotropic state was reached with a slower dynamic, yielding a long‐lasting disordered configuration with random alignment and weak cell anisotropy (Figure S5 and Video S6, Supporting Information). While dielectrically anisotropic nematic liquid crystals could eventually evolve to the alternative stable mode upon system perturbation, the lack of coherent cell organization in presence of flow weakened the intercellular adhesion capability, exposing the endothelium to a non‐evolving, disruptive transition and marking an important difference with the liquid crystal case.

**Figure 5:**
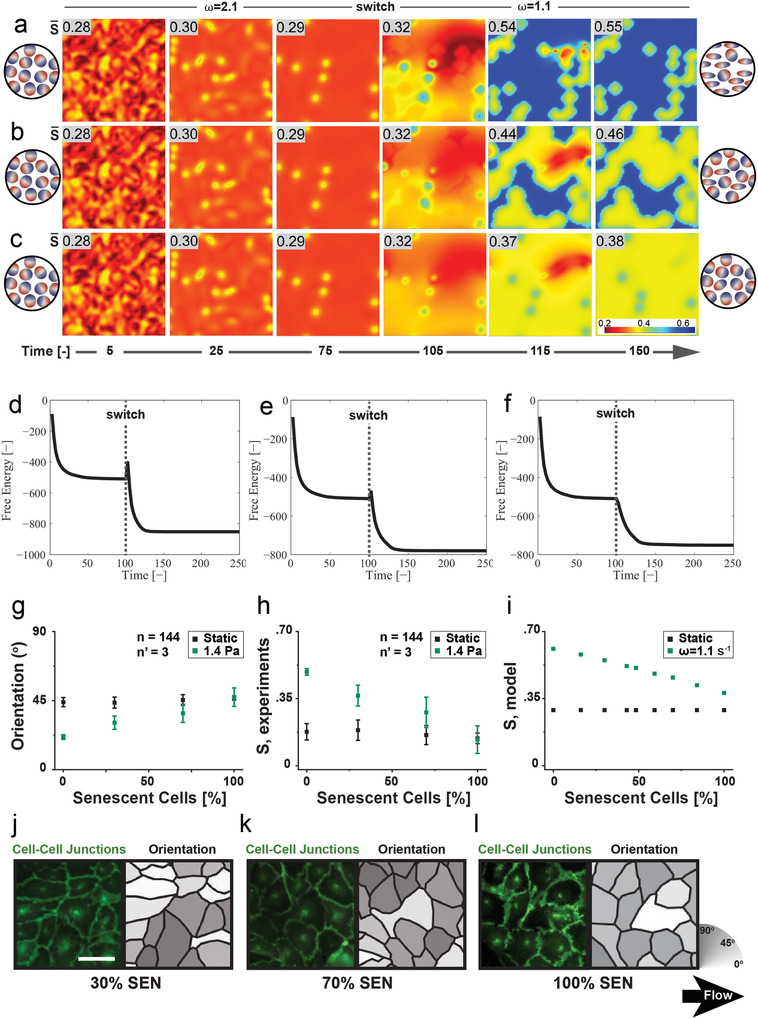
System transitions in a mixed population of rod‐like and disk‐like nematic elements (a‐f, i: in silico, g,h,j‐l: in vitro). a) Heat maps of the transient nematic order parameter, *S*, for polar nematic crystal with 30% disk‐like crystals in the presence of an oscillatory electric field upon switching frequency from *ω*_0_ to *ω*_L_ (the mean values, $\bar{S},$ are inset). b) Corresponding heat maps of *S* for 70% disk‐like crystals, that is, from *ω*_0_ to *ω*_L_. A corresponding representation of the initial (left) and final (right) states is displayed in the circular cartoons. c) Corresponding heat maps of *S* for 100% disk‐like crystals, that is, from *ω*_0_ to *ω*_L_. A corresponding representation of the initial (left) and final (right) states is displayed in the circular cartoons. d) Free energy development during the system transition depicted in (a). e) Corresponding free energy development during the transition depicted in (b). f) Corresponding free energy development during the transition depicted in (c). g) Orientation angle in static conditions and after 8–16 h of 1.4 Pa WSS, with control endothelial cells (0% SEN, 8 h), mixed population of control and senescent endothelial cells (30% SEN and 70% SEN, 16 h) and senescent endothelial cells (100% SEN, 16 h). h) Order parameter, *S*, in static conditions and after 8–16h of 1.4 Pa WSS for control endothelial cells (0% SEN, 8 h), mixed population of control and senescent endothelial cells (30% SEN and 70% SEN, 16 h) and senescent endothelial cells (100% SEN, 16 h). i) Order parameter, *S*, model simulation results in static conditions and *ω* = 1.1s^−1^, for control endothelial cells (0% SEN, 0% disk‐like nematic particles), mixed population of control and senescent endothelial cells and senescent endothelial cells (100% SEN, 100% disk‐like nematic particles). Representative fluorescent images of cell‐cell junctions (VE‐Cadherin or *β*‐catenin) distribution (green) at cell‐to‐cell junctions (left panels in (j–l)) and corresponding cell profiles with color‐coded cell orientation (right panels in (j–l)) as encoded by corresponding color scale bar for mixed population of control and senescent endothelial cells (30% SEN and 70% SEN, 16 h) and senescent endothelial cells (100% SEN, 16 h). Scale bar is 50 µm. The number of analyzed cells is reported as n and the number of independent experiments as n’. Line bars depict the mean and error bars indicate the standard deviation.

Senescent endothelial cells fail to change shape in response to flow^[^ ^37^ ^]^ and generate monolayers where the cell aspect ratio is unaffected, while the cell orientation remains randomly distributed despite the exposure to WSS (**Figure** **5**). The functional decay of senescent cells was modeled by including particles that do not change shape in response to external fields in the in silico representation, which we term senescent particles. In particular, elements featuring *B* = +1 (disk‐like nematic, see Supplementary Information, section “Modeling Nematic Liquid Crystals”) were introduced, in contrast to elements with *B* = −1 (rod‐like nematic) representative of the control, healthy and young endothelial cells. In agreement with the experimental results, systems of senescent particles, did not evolve to an ordered state upon exposure to the external field (Figure 5a–c).

Based on this analogy, the model was inquired on the effect of a growing fraction of senescent elements in an otherwise nematic system. This was obtained by systematically varying the ratio between the two types of particles and retrieving the system ordering at steady state. Figure 5i shows the system's transition with increasing population of the senescent particles. Corresponding free energy evolutions Figure 5d–f shows the typical transition of the system, indicative of the field‐induced reorientation gradually waning while the average order parameter of the system at steady state decreases monotonously with increasing fraction of senescent particles (Figure 5i).

The model prediction was experimentally verified exposing endothelial monolayers, featuring precisely controlled ratios of young and senescent cells, to flow generating a WSS of 1.4 Pa (Figure 5j–l). The average cell orientation along the direction of flow was gradually lost in monolayers featuring 0, 30, 70, and 100% of senescent endothelial cells (≈21°, 31°, 37°, and 47°, respectively). The results therefore exhibited excellent qualitative agreement with the simulation, indicating that the response to flow is partially maintained as long as a fraction of young and healthy cells are present in the tissue.

## 3 Discussion and Conclusions

Coordinated movement of epithelial sheets, observed during tissue morphogenesis and regeneration, manifests itself through the collective organization of cell shape, orientation, and migration. The explanation of these complex activities can be facilitated by rationally adapting physical models developed for dense systems of anisotropic particles displaying nematic ordering.^[^ ^1^, ^38^ ^]^ This analogy extends to capture specific tissue properties, such as the emergence of local topological defects that, in mature tissues, enable the control of global density and homeostasis.^[^ ^7^, ^39^ ^]^ Adult epithelia retain a functional level of plasticity to respond to external mechanical stimuli, whereby tissue fluidity can be transiently reactivated adapting to a dynamic physical environment.^[^ ^40^ ^]^ At the fundamental level, subtle variations of cell shape and junctional tension can stir the system towards a pathological response, as observed in asthma^[^ ^40^ ^]^ and cancer.^[^ ^41^ ^]^ Exploiting the analogy with inanimate systems, in silico models are able to reproduce these maladaptive processes and individuate their key control parameters.

Much of this largely unexpected behavior can also manifest itself as collective coordination in endothelial ensembles, naturally developed to interact with a mechanical environment dominated by hemodynamic stress loads. Onset of autonomous circulation at birth exposes the vasculature to a complex mechanical landscape, in which the tissue must develop to serve a growing body. The control of shape changes and cell orientation renders a vascular tree, which radiates larger vessels from the heart and tapers in the body periphery. Yet, the intrinsic endothelial plasticity proves insufficient to adapt to local flow perturbations, such as those induced by stenosis or deployment of cardiovascular implants. Excessive flow intensity increase or decrease represent hotspots for inflammatory processes mining the stability of endothelial junctions,^[^ ^14^ ^]^ and therefore of the overall monolayer. These are locations where endothelial function is gradually lost, and the typical traits of cell senescence arise. Senescent vascular tissues are prone to denudation, which precedes the development of atherosclerotic lesions.^[^ ^42^ ^]^

Such behavior generates important and extremely interesting fundamental questions, such as, why does a distinctive global order emerge in different locations of the vasculature? Why do adult endothelia fail to adapt to variations of flow intensity? Or, how does the appearance of senescent cells in an otherwise healthy monolayer affect the collective tissue response to flow? It is clear that the physical description of endothelial monolayers poses new challenges, which go beyond the capabilities of models developed for epithelial tissues. The explanation lies in the unique nature of the stimulus instructing endothelial adaptation, a vector quantity encoding at once directionality, which drives cell polarization, and intensity, for structural remodeling. These are distinct areas of information that can be processed independently by endothelial tissues, yielding specific multicellular configurations.

The analogy with externally driven dual‐frequency liquid crystals associates an external stimulus to the change in the dielectric anisotropy and resolves nematic states with orthogonal collective alignment to the frequency of an external electric field, but identical polarization. On the resulting phase diagram, the transition between the two distinct ordered states crosses an intermediate phase characterized by high levels of disorder. When this model is applied to describe endothelial systems, it predicts the emergence of a random tissue configuration upon variations of flow intensity, which persists experimentally to the point where the structural integrity of the monolayer is disrupted, before another ordered state is reached. Table SI summarizes the relevant parameters responsible for triggering the in vitro and in silico order‐transition of the system.

Based on the proposed physical description, the catastrophic event observed in endothelial tissues may not be considered as a maladaptive response per se, and thus rooted in the downstream biological signaling, but rather as an intrinsic property of a system that was once primed by an external anisotropic field, loses plasticity and adaptability. Along this line of thinking, the pathological trigger would be encoded by the variation of local hemodynamics. Endothelial tissues may only be able to adapt once, but not several times.

The biological phenomenon of senescence subtends to the loss of endothelial cell adaptivity to flow.^[^ ^37^ ^]^ Individual endothelial cells drift to functional decay, driven by the timely accumulation of genetic and metabolic damage.^[^ ^24^ ^]^ This could lead to a progressive impairment of the collective response and functionality in tissues populated by a growing number of senescent cells. An alternative hypothesis would see the maintenance of complete (or almost complete) adaptivity to flow until the percentage of old cells remains below a functional threshold. In this scenario, as the limit is passed, the system collapses and fully loses its responsiveness to the external field.

The emergence of senescent cells is captured by the proposed model through the introduction of senescent elements. The parallelism with the physical system predicts a linear decrease of the collective ordering in response to the external field along with a growing fraction of senescent particles. These results assume that nematic and senescent neighboring elements do not influence each other. The excellent agreement of the model forecast with the experimental validation, indicates that the topology of the biological assembly does not influence the overall multicellular response. This translates into a limited or absent biological and physical communication between adjacent senescent and young endothelial cells. Cell‐to‐cell junctions establish this communication in healthy endothelial tissues and may be progressively lost when they are exposed to noxious stimuli inducing senescence.

## 4 Experimental Section

### Cell Culture, Substrate Coating, and Seeding

Primary human umbilical vein endothelial cells (HUVECs; Invitrogen, USA) were grown in medium 200PRF supplemented with fetal bovine serum 2% v/v, hydrocortisone 1 mg mL^−1^, human epidermal factor 10 ng mL^−1^, basic fibroblast growth factor 3 ng mL^−1^ and heparin 10 mg mL^−1^ (all reagents from Invitrogen), and were incubated at 37 °C and 5% CO_2_. All reported experiments were performed using cells with less than six passages in vitro.

Silicone substrates were incubated in a custom‐built vacuum oven at 90 °C for 4 h, washed 1 min in methanol, and incubated for another 2 h in the vacuum to remove ligands. They were subsequently coated with 1,5% gelatin (104 070, Merck Millipore, USA). Finally, the surfaces were washed twice with warm PBS before applying medium and seeding cells at desired concentrations. To generate a confluent monolayer, cells were seeded on the surfaces at high density (3.5–5×10^4^ cell cm^−2^) and cultured for three days (Figure S1, Supporting Information).^[^ ^43^ ^]^ In developing endothelial monolayers, cell motility decreased and collective motion emerged as a function of local density (Video S1, Supporting Information). However, even upon proliferation arrest, endothelial cells continued to be motile and exchange neighbors (Figure S1, Supporting Information).

### In Vitro Induced Senescence

Endothelial senescence was induced in vitro by applying a previously described protocol.^[^ ^44^ ^]^ Briefly, 24 h after seeding low passage HUVECs at an initial density of 8 × 10^3^ cells cm^−2^, cells were treated with TNF‐*α* (10 ng mL^−1^, #300‐01A, PeproTech, USA) for 6 days, followed by 3 days of recovery in full growth medium. Exchange of medium with TNF‐a was performed every other day to obtain senescent cells.

To obtain monolayers featuring a controlled fraction of senescent cells, endothelial cells from the two populations (young or senescent) were detached from their respective cultures, counted, and mixed to the desired ratio (70:30 and 30:70; Figure 5). The resulting cell suspension was then seeded to generate a confluent monolayer for flow experiments, as previously described.

### cTFM

The two components of CY52‐276 polydimethylsiloxane (Dow Corning) and 0.05% (v/v) poly(dimethylsiloxane‐b‐ethylene oxide; Polysciences) were mixed thoroughly at a ratio of 9:10 (A:B) which provides an elastic modulus of ≈12.6 kPa^[^ ^27^ ^]^ for 5 min, degased for 2 min, and spin‐coated on 170 µm thick cyclic olefin copolymer (COC) for 1 min at 1500 rpm to achieve a target thickness of ≈35 µm. The silicone was then cured at 70 °C for 30 min. Substrates were then maintained in a clean, dust‐free, and dry environment to prevent fouling until use. To avoid aging, samples were used 2 weeks after fabrication.

Arrays of red quantum dot (QD) nanodiscs were deposited on the substrate by electrohydrodynamic (EHD) nanodrip printing^[^ ^45^ ^]^ with a spacing of 5 µm, as previously reported.^[^ ^27^ ^]^ Briefly, colloidal QD ink was ejected onto the silicone substrate from a micro‐sized gold‐coated nozzle by an electric field. The relative position of nozzle and sample, as well as the electric field, can be modulated to control the printing process. The EHD printing technology can be commercially obtained through an ETH Zurich spin‐off company (www.scrona.ch).

### Flow Experiments

A custom‐designed parallel plate flow chamber was used to apply a unidirectional laminar flow yielding a constant shear stress of 1.4 or 8 Pa to endothelial monolayers as reported previously.^[^ ^19^, ^46^ ^]^

To analyze the role of VEC phosphorylation, the Src inhibitor PP1 (Merck Millipore, 10µm) was added to the culture medium 1 h prior to WSS switch from 8 to 1.4 Pa as previously reported.^[^ ^14^ ^]^

For live PCP analysis, endothelial cells were incubated for 16 h at 37 °C with the CellLight Golgi RFP (Bac Mam, Invitrogen) for visualization of the Golgi apparatus (2 µL/10^4^ cell in media). Media was then exchanged and cells were stained with NucBlue (2 drops mL^−1^ of media; NucNlue Live ReadyProbes Reagent, R37605, ThermoFisher) 35 min before the experiment. The substrates supporting fluorescently labeled monolayers were then placed mounted in the flow bioreactor.

### Antibodies

The following primary antibodies were used: goat polyclonal anti‐VE‐cadherin (1:200, sc‐6458, Santa Cruz Biotechnology), anti‐*β*‐catenin (1:100, #610 154, BD Transduction Laboratories), rabbit polyclonal anti‐pY658‐VEC^[^ ^14^ ^]^ (1µg mL^−1^), and mouse monoclonal anti‐58K Golgi protein (1:100, ab27043, Abcam). The secondary antibodies were donkey anti‐goat‐Alexa 488 (Invitrogen, A‐11055), donkey anti‐Mouse‐Alexa 555 (Invitrogen, A‐31570), chicken anti‐rabbit‐Alexa 647 (Invitrogen, A‐21443), and donkey anti‐mouse‐Alexa 488 (Invitrogen, #A21202).

### Immunostaining

HUVECs were fixed for 20 min with 2% paraformaldehyde (PFA) at room temperature. Next, the cells were permeabilized with 1% Triton x‐100 in PBS for 5 min. After washing the samples three times for 5 min with PBS, they were incubated in 5% w/v bovine serum albumin (Sigma‐Aldrich, USA) in PBS for 2 h at room temperature. The samples were incubated with the respective antibodies (See Antibodies section) or with TRITC‐phalloidin (Sigma Aldrich), overnight at 4 °C.

Subsequently, the samples were rinsed 2 times for 30 min with PBS and then 2 times for 30 min with 5% BSA in PBS. They were then incubated with the corresponding secondary antibodies for 45 min at room temperature. Finally, the samples were washed 4 times for 30 min with PBS. For staining of nuclei, Hoechst was added at 10 µg mL^−1^ during a washing step.

### Cell Microscopy

Cell adaptation to flow was monitored using an inverted Nikon‐Ti wide‐field microscope (Nikon, Japan) and an incubation chamber (Life Imaging Services, Switzerland). Both the flow bioreactor and the medium reservoir were maintained at a controlled temperature of 37 °C and CO_2_ concentration of 5%. Images were collected with a 20x, 0.45 NA long‐distance objective (Plan Fluor, Nikon, Japan). Time‐lapse experiments were set to routinely collect images, in different spatial positions of the sample, in the DAPI (nuclei) and TRITC (Golgi apparatus or fluorescent QDs) channel with a time resolution of 20 or 30 min.

VEC, Actin, and cell nuclei distribution were acquired in immunostained samples using a 60X, 1.4 NA oil immersion objective (Plan Fluor, Nikon, Japan), and the FITC, TRITC, and DAPI filter; respectively. Samples were imaged with an inverted Nikon‐Ti spinning disk confocal microscope (Nikon, Japan) equipped with an Andor DU‐888 camera (Oxford Instruments, UK) and a pE‐100 LED illumination system (CoolLED Ltd, Andover, United Kingdom).

### Modeling of Nematic System Transitions

A publicly available, previously published algorithm was adapted and used for modeling the nematic system transitions^[^ ^33^ ^]^ under an electric field with constant amplitude *E* (= [*E_x_*,*E_y_*,*E_z_*] = [1, 0, 0] Vm^−1^). Dual‐frequency nematic particles were then employed, where the magnitude and sign of the dielectric anisotropy (Δ*ε*) depends on the frequency of the imposed electric field, *ω*,^[^ ^35^ ^]^ a property previously reported.^[^ ^47^ ^]^ Both low‐*ω* (dipole moment along the long molecular axis, Δ*ε* > 0) and high‐*ω* (dipole moment along the short molecular axis, Δ*ε* < 0) responses were explored, with an intermediate *ω* corresponding to Δ*ε* = 0. What followed below was a brief summary of the model and the parameters used in the simulations (see ref. [33] for full details).

The orientational order of the nematic liquid crystal was given by the tensor *Q*. For a uniaxial system whose molecular orientation was defined by the unit vector *u*, written as, $Q\equiv S(nn^{T}-\frac{1}{3}I)$, where $S=\left\langle \mathrm{co}s^{2}\theta \right\rangle -\frac{1}{3}$ is the scalar order parameter, *n* is the director (in nematic liquid crystals, the rod‐like molecules orient themselves in space along an arbitrary direction called the director), *I* is the identity matrix, and *θ* is the angle of the rods with respect to the director (cos *θ* = *n* · *u*).

### Image Analysis

The measurement of individual cell orientation along a chosen direction and aspect ratio required the manual drawing of the cell profile. This was experimentally obtained from fluorescent images reporting the distribution of VE‐cadherin or *β*‐catenin. The method was equivalent to the well‐validated approach for the segmentation of epithelial cells in corresponding monolayers, based on the distribution of the junctional protein ZO‐1.^[^ ^48^ ^]^

The “Freehand selection” tool of FiJi^[^ ^49^, ^50^ ^]^ was used to identify the borders of each cell and create a region of interest (ROI) corresponding to the cell perimeter. A custom‐made algorithm implemented in MATLAB was then used to fit an ellipse to each cell's ROI. The cell aspect ratio was then defined as the ratio between the major and the minor axis of the fitted ellipse. Based on this definition, the aspect ratio acquires values of 1 for a perfect circle (i.e., no cellular elongation) or larger than 1 for increasing degrees of cell shape elongation. From the same ROI the cell orientation was defined as the angle between the fitted ellipse major axis and the chosen reference direction. For cells exposed to flow, the reference corresponds to the flow direction. In static conditions the horizontal direction was arbitrarily selected as reference. Based on the system symmetry cells acquire orientation values ranging from 0° (for perfect alignment with the reference direction) to 90° (for orthogonal alignment to the reference direction).

In fully isotropic settings, such as in static conditions,^[^ ^19^, ^51^, ^52^, ^53^ ^]^ individual cells maintain randomly distributed alignment. In this context, the mathematical average of measured orientation values was close to 45°. Any deviation from this midpoint indicates a level of collective orientation. When cells were exposed to flow, they collectively align along its direction in a process requiring coordinated migration and cell shape remodeling.^[^ ^51^, ^52^, ^53^ ^]^ This process was reflected by a decrease of the average alignment to values closer to 0°. Similarly, a coordinated alignment in the direction perpendicular to flow leads to an average increase of global alignment to values closer to 90°.^[^ ^18^, ^21^ ^]^ The average orientation value alone does not describe the extent of collective cell alignment. It must be complemented by the width of datapoint distribution around the average, which was defined by the standard deviation of the mean (SD) (Figure S12, Supporting Information). The measurement of average orientation and the corresponding SD in static samples, the horizontal axis of the image was arbitrarily selected as reference.

To compare the experimental results with the simulation data, the order parameter for the experiments was defined as $S=\left\langle \mathrm{co}s^{2}\theta \right\rangle -\frac{1}{3}$, where *θ* is the orientation angle.

The evolution of PCP in monolayers was monitored using a custom‐developed algorithm utilizing Imaris and MATLAB (Figure S3, Supporting Information). First, fluorescent time‐lapse images of the cell nuclei and Golgi were imported in Imaris (Oxford Instruments, UK). Structures were individuated using the spot detection frame‐wise in Imaris. The coordinates of the cellular structures were then imported to MATLAB, where they were processed with the following protocol: from the detected nuclei a Voronoi tessellation was generated. Detected Golgi that were located within a Voronoi cell of a nucleus, were associated to that respective nucleus. For cases that more than one Golgi was detected, the final position was determined by averaging their weighted positions. The weight for the positions decreased with increasing distance from the nucleus. Last, a vector from the center of the nucleus to the weighted Golgi position was created. The vector's angle with the direction of flow (*ϕ*) was then used to calculate the PCP index (cos *ϕ*).

Moving the Golgi towards a specific direction allows the cells to organize specific activities in an asymmetric fashion. Most notably, directional migration required the continuous flow of membrane components to allow for shape changes.^[^ ^54^ ^]^ PCP was accomplished by the endothelial ensembles in response to a directional stimulus. This way cells can migrate collectively against the flow direction, that was upstream.^[^ ^55^ ^]^ This notation was relevant for the reference to vascular physiology in the mammalian body, whereas endothelial cells migrate along the arterial vessels towards the heart. The opposite PCP (i.e., downstream), would subside to a migration along the flow direction, which was indicative of loss of tissue integrity and precedes denudation of the substrate.^[^ ^56^ ^]^

For the migration experiments, the nuclei were initially detected using Imaris (Oxford Instruments, UK). The position of the nuclei over time was then tracked in MATLAB using a custom‐made algorithm. The lower detection limit for actual migration was set to 10 µm h^−1^, below which the cells were considered stationary. Data for migration velocity were automatically extracted using MATLAB.

Pathfinding measures were performed using cell tracking software Imaris (Oxford Instruments, UK). Time‐lapse videos were uploaded into Imaris, and the voxel size and time interval were adjusted before particle tracking. The velocity and density of the cells were obtained by tracking the migration of individual cells over time until confluency.

Cell image velocimetry (CIV)^[^ ^57^ ^]^ toolbox was used for the correlation length calculation. The velocity fields (u and v) in the two directions (x and y) were provided by the CIV analysis.

The magnitude of the velocity *M* was calculated with MATLAB using the velocity fields: *M* = (u(x,y)^2^ + v(x,y)^2^))^1/2^. For all purposes, the mean velocity was subtracted from the calculated velocity field to avoid any drift‐related bias and to get the fields.

To estimate the distance over which movements were correlated, the velocity spatial correlation function C_vv_ was calculated. The velocity correlation function C_vv_ was fitted with a decreasing exponential function of the form f(r) = e^−(r/CL)^ in order to extract the velocity correlation length C_L_.

Traction forces were calculated frame‐by‐frame utilizing the cTFM software.^[^ ^27^ ^]^ The detection and meshing were done in MATLAB, as described previously.^[^ ^27^ ^]^ For the calculation of intercellular stresses, the protocol suggested by Tambe et al.^[^ ^30^ ^]^ was used. According to this, for each spatial and temporal position, the intracellular stresses were calculated. The total strain energy was calculated according to Butler et al.^[^ ^58^ ^]^ by integrating over the surface the dot product of tractions and displacements.

For the colocalization analysis, the Pearson's coefficient was extracted from each image stack using the colocalization section of Imaris (Oxford Instruments, UK). Before the colocalization analysis, the “Background Subtraction” function of Imaris was applied to both the blue and red channels. During the colocalization analysis, threshold values calculated based on^[^ ^59^ ^]^ were imposed for both channels.

### Proliferation Assay

The DNA synthesis–based cell proliferation assay was performed using a commercially‐available Click‐iT EdU Imaging Kits Protocol (Thermo Fisher Scientific) and following the manufacturer's recommendations. Endothelial cells were seeded on the substrates and incubated overnight with 10 µm 5‐Ethynyl‐2'‐deoxyuridine (EdU) labeling solution before the fixing (day 0, 1, 2, 3, and 6). After fixation with 4% formaldehyde and permeabilization with 0.5% Triton X‐100 in PBS, samples were stained and imaged using a fluorescence microscope.

### Statistical Analysis

The Shapiro–Wilk test was used to test for the normality of data. For non‐normal distributed data, Mann–Whitney U test or Wilcoxon signed‐ranked test was performed. Boxes in all box plots extend from the 25th to the 75th percentiles, with a line at the median and a square representing the mean. Whiskers extend to 1.5x IQR (inter‐quantile range) or the max/min data points if they fall within 1.5xIQR. Line graphs depicted the mean with error bars for the standard deviation. The total number of events counted was shown in the graphs. The number of independent experiments was reported as n’ and the number of total fields of view or cells analyzed was shown as n.

## Conflict of Interest

The authors declare no conflict of interest.

## Author Contributions

G.S and T.L. contributed equally to the work. T.L., G.S., A.F., and D.P. designed the study; G.S., C.G., and N.C. performed the flow chamber experiments. T.L. developed algorithms. T.L., G.S., and N.C. performed the analysis. T.S., T.R., T.L., R.C., and F.G. adapted, ran, and evaluated the simulations. A.F. wrote the first draft of the manuscript. All authors gave input at various stages during writing.

## Supporting information

Supporting Information

Supplemental Video 1

Supplemental Video 2

Supplemental Video 3

Supplemental Video 4

Supplemental Video 5

Supplemental Video 6

## Data Availability

The data that support the findings of this study are available from the corresponding author upon reasonable request.

## References

[1] A. Mongera , P. Rowghanian , H. J. Gustafson , E. Shelton , D. A. Kealhofer , E. K. Carn , F. Serwane , A. A. Lucio , J. Giammona , O. Campas , Nature 2018, 561, 401. 30185907 10.1038/s41586-018-0479-2 PMC6148385

[2] X. Trepat , M. R. Wasserman , T. E. Angelini , E. Millet , D. A. Weitz , J. P. Butler , J. J. Fredberg , Nat. Phys. 2009, 5, 426.

[3] B. A. Camley , W. J. Rappel , J. Phys. D: Appl. Phys. 2017, 50, 113002. 28989187 10.1088/1361-6463/aa56fe PMC5625300

[4] B. Ladoux , R. M. Mege , Nat. Rev. Mol. Cell Biol. 2017, 18, 743. 29115298 10.1038/nrm.2017.98

[5] T. B. Saw , W. Xi , B. Ladoux , C. T. Lim , Adv. Mater. 2018, 30, e180257. 10.1002/adma.201802579 30156334

[6] X. Trepat , E. Sahai , Nat. Phys. 2018, 14, 671.

[7] T. B. Saw , A. Doostmohammadi , V. Nier , L. Kocgozlu , S. Thampi , Y. Toyama , P. Marcq , C. T. Lim , J. M. Yeomans , B. Ladoux , Nature 2017, 544, 212. 28406198 10.1038/nature21718 PMC5439518

[8] G. Duclos , S. Garcia , H. G. Yevick , P. Silberzan , Soft Matter 2014, 10, 2346. 24623001 10.1039/c3sm52323c

[9] L. Wagstaff , M. Goschorska , K. Kozyrska , G. Duclos , I. Kucinski , A. Chessel , L. Hampton‐O'Neil , C. R. Bradshaw , G. E. Allen , E. L. Rawlins , P. Silberzan , R. E. Carazo Salas , E. Piddini , Nat. Commun. 2016, 7, 1137. 10.1038/ncomms11373 PMC4848481 27109213

[10] G. Duclos , C. Blanch‐Mercader , V. Yashunsky , G. Salbreux , J. F. Joanny , J. Prost , P. Silberzan , Nat. Phys. 2018, 14, 728. 30079095 10.1038/s41567-018-0099-7 PMC6071846

[11] C. Wang , B. M. Baker , C. S. Chen , M. A. Schwartz , Arterioscler., Thromb., Vasc. Biol. 2013, 33, 2130. 23814115 10.1161/ATVBAHA.113.301826 PMC3812824

[12] E. Dejana , Nat. Rev. Mol. Cell Biol. 2004, 5, 261. 15071551 10.1038/nrm1357

[13] V. L. Bautch , K. M. Caron , Cold Spring Harbor Perspect. Biol. 2015, 7, a00826. 10.1101/cshperspect.a008268 PMC4355271 25731762

[14] F. Orsenigo , C. Giampietro , A. Ferrari , M. Corada , A. Galaup , S. Sigismund , G. Ristagno , L. Maddaluno , G. Y. Koh , D. Franco , V. Kurtcuoglu , D. Poulikakos , P. Baluk , D. McDonald , M. G. Lampugnani , E. Dejana , Nat. Commun. 2012, 3, 1208. 23169049 10.1038/ncomms2199 PMC3514492

[15] H. B. Kwon , S. Wang , C. S. Helker , S. J. Rasouli , H. M. Maischein , S. Offermanns , W. Herzog , D. Y. Stainier , Nat. Commun. 2016, 7, 1180. 10.1038/ncomms11805 PMC4895482 27248505

[16] W. W. Sugden , R. Meissner , T. Aegerter‐Wilmsen , R. Tsaryk , E. V. Leonard , J. Bussmann , M. J. Hamm , W. Herzog , Y. Jin , L. Jakobsson , C. Denz , A. F. Siekmann , Nat. Cell Biol. 2017, 19, 653. 28530658 10.1038/ncb3528 PMC5455977

[17] J. A. Ukropec , M. K. Hollinger , M. J. Woolkalis , Exp. Cell Res. 2002, 273, 240. 11822879 10.1006/excr.2001.5453

[18] M. A. Ostrowski , N. F. Huang , T. W. Walker , T. Verwijlen , C. Poplawski , A. S. Khoo , J. P. Cooke , G. G. Fuller , A. R. Dunn , Biophys. J. 2014, 106, 366. 24461011 10.1016/j.bpj.2013.11.4502 PMC3907231

[19] F. Robotti , D. Franco , L. Banninger , J. Wyler , C. T. Starck , V. Falk , D. Poulikakos , A. Ferrari , Biomaterials 2014, 35, 8479. 25017097 10.1016/j.biomaterials.2014.06.046

[20] S. McCue , D. Dajnowiec , F. Xu , M. Zhang , M. R. Jackson , B. L. Langille , Circ. Res. 2006, 98, 939. 16527990 10.1161/01.RES.0000216595.15868.55

[21] J. T. Butcher , A. M. Penrod , A. J. Garcia , R. M. Nerem , Arterioscler., Thromb., Vasc. Biol. 2004, 24, 1429. 15117733 10.1161/01.ATV.0000130462.50769.5a

[22] J. J. Chiu , S. Chien , Physiol. Rev. 2011, 91, 327. 21248169 10.1152/physrev.00047.2009 PMC3844671

[23] T. Z. Nazari‐Shafti , J. P. Cooke , Methodist Debakey Cardiovasc. J. 2015, 11, 172. 26634025 10.14797/mdcj-11-3-172 PMC4666424

[24] A. J. Donato , R. G. Morgan , A. E. Walker , L. A. Lesniewski , J. Mol. Cell. Cardiol. 2015, 89, 122. 25655936 10.1016/j.yjmcc.2015.01.021 PMC4522407

[25] K. B. Vartanian , M. A. Berny , O. J. McCarty , S. R. Hanson , M. T. Hinds , Am. J. Physiol. Cell Physiol. 2010, 298, C333. 19923423 10.1152/ajpcell.00340.2009

[26] D. Franco , M. Klingauf , M. Bednarzik , M. Cecchini , V. Kurtcuoglu , J. Gobrecht , D. Poulikakos , A. Ferrari , Soft Matter 2011, 7, 7313.

[27] M. Bergert , T. Lendenmann , M. Zundel , A. E. Ehret , D. Panozzo , P. Richner , D. K. Kim , S. J. Kress , D. J. Norris , O. Sorkine‐Hornung , E. Mazza , D. Poulikakos , A. Ferrari , Nat. Commun. 2016, 7, 1281. 10.1038/ncomms12814 PMC5056408 27681958

[28] C. Malinverno , S. Corallino , F. Giavazzi , M. Bergert , Q. S. Li , M. Leoni , A. Disanza , E. Frittoli , A. Oldani , E. Martini , T. Lendenmann , G. Deflorian , G. V. Beznoussenko , D. Poulikakos , K. H. Ong , M. Uroz , X. Trepat , D. Parazzoli , P. Maiuri , W. M. Yu , A. Ferrari , R. Cerbino , G. Scita , Nat. Mater. 2017, 16, 587. 28135264 10.1038/nmat4848 PMC5407454

[29] M. Panagiotakopoulou , T. Lendenmann , F. M. Pramotton , C. Giampietro , G. Stefopoulos , D. Poulikakos , A. Ferrari , Mol. Biol. Cell 2018, 29, 2528. 30113874 10.1091/mbc.E17-12-0726 PMC6254576

[30] D. T. Tambe , C. C. Hardin , T. E. Angelini , K. Rajendran , C. Y. Park , X. Serra‐Picamal , E. H. Zhou , M. H. Zaman , J. P. Butler , D. A. Weitz , J. J. Fredberg , X. Trepat , Nat. Mater. 2011, 10, 469. 21602808 10.1038/nmat3025 PMC3135682

[31] R. Steward Jr. , D. Tambe , C. C. Hardin , R. Krishnan , J. J. Fredberg , Am. J. Physiol. Cell Physiol. 2015, 308, C657. 25652451 10.1152/ajpcell.00363.2014 PMC4398851

[32] F. Giavazzi , M. Paoluzzi , M. Macchi , D. P. Bi , G. Scita , M. L. Manning , R. Cerbino , M. C. Marchetti , Soft Matter 2018, 14, 3471. 29693694 10.1039/c8sm00126j PMC5995478

[33] B. F. de Oliveira , P. P. Avelino , F. Moraes , J. C. Oliveira , Phys. Rev. E: Stat., Nonlinear, Soft Matter Phys. 2010, 82, 04170. 10.1103/PhysRevE.82.041707 21230295

[34] P. J. W. Debye , Polar molecules, Dover publications, New York 1929.

[35] H. Xianyu , S.‐T. Wu , C.‐L. Lin , Liq. Cryst. 2009, 36, 717.

[36] P. J. Wojtowicz , P. Sheng , E. Priestley , Introduction to liquid crystals, Springer, Boston, MA 1975.

[37] N. Chala , S. Moimas , C. Giampietro , X. Zhang , T. Zambelli , V. Exarchos , T. Z. Nazari‐Shafti , D. Poulikakos , A. Ferrari , Nano Lett. 2021, 21, 4911. 34081865 10.1021/acs.nanolett.1c00064

[38] E. H. Barriga , K. Franze , G. Charras , R. Mayor , Nature 2018, 554, 523. 29443958 10.1038/nature25742 PMC6013044

[39] K. Kawaguchi , R. Kageyama , M. Sano , Nature 2017, 545, 327. 28403137 10.1038/nature22321

[40] J. A. Park , J. A. Mitchel , N. T. Qazvini , J. H. Kim , C. Y. Park , J. P. Butler , E. Israel , S. H. Randell , S. A. Shore , J. M. Drazen , J. J. Fredberg , Ann. Am. Thorac. Soc. 2016, 13, S10. 10.1513/AnnalsATS.201506-382MG PMC5015726 27027941

[41] A. Palamidessi , C. Malinverno , E. Frottoli , S. Corallino , E. Barbieri , S. Sigismund , P. P. Di Fiore , G. V. Beznoussenko , E. Martini , M. Garrè , D. Parazzoli , I. Ferrara , C. Tripodo , F. Giavazzi , R. Cerbino , G. Scita , Nat. Mater. 2019, 18, 1252. 31332337 10.1038/s41563-019-0425-1

[42] B. C. Berk , Circulation 2008, 117, 1082. 18299513 10.1161/CIRCULATIONAHA.107.720730

[43] M. G. Lampugnani , M. Corada , P. Andriopoulou , S. Esser , W. Risau , E. Dejana , J. Cell Sci. 1997, 110, 2065. 9378757 10.1242/jcs.110.17.2065

[44] S. Y. Khan , E. M. Awad , A. Oszwald , M. Mayr , X. Yin , B. Waltenberger , H. Stuppner , M. Lipovac , P. Uhrin , J. M. Breuss , Sci. Rep. 2017, 7, 3950. 28045034 10.1038/srep39501 PMC5206708

[45] P. Galliker , J. Schneider , H. Eghlidi , S. Kress , V. Sandoghdar , D. Poulikakos , Nat. Commun. 2012, 3, 89. 10.1038/ncomms1891 22692533

[46] G. Stefopoulos , F. Robotti , V. Falk , D. Poulikakos , A. Ferrari , Small 2016, 12, 4113. 27346806 10.1002/smll.201503959

[47] H. Bücher , R. Klingbiel , J. VanMeter , Appl. Phys. Lett. 1974, 25, 186.

[48] S. Lohmann , C. Giampietro , F. M. Pramotton , D. Al‐Nuaimi , A. Poli , P. Maiuri , D. Poulikakos , A. Ferrari , Adv. Sci. 2020, 7, 200121. 10.1002/advs.202001213 PMC7404176 32775171

[49] J. Schindelin , I. Arganda‐Carreras , E. Frise , V. Kaynig , M. Longair , T. Pietzsch , S. Preibisch , C. Rueden , S. Saalfeld , B. Schmid , J. Y. Tinevez , D. J. White , V. Hartenstein , K. Eliceiri , P. Tomancak , A. Cardona , Nat. Methods 2012, 9, 676. 22743772 10.1038/nmeth.2019 PMC3855844

[50] C. A. Schneider , W. S. Rasband , K. W. Eliceiri , Nat. Methods 2012, 9, 671. 22930834 10.1038/nmeth.2089 PMC5554542

[51] D. Franco , F. Milde , M. Klingauf , F. Orsenigo , E. Dejana , D. Poulikakos , M. Cecchini , P. Koumoutsakos , A. Ferrari , V. Kurtcuoglu , Biomaterials 2013, 34, 1488. 23182348 10.1016/j.biomaterials.2012.10.007

[52] M. J. Levesque , R. M. Nerem , J. Biomech. Eng. 1985, 107, 341. 4079361 10.1115/1.3138567

[53] A. M. Malek , S. Izumo , J. Cell Sci. 1996, 109, 713. 8718663 10.1242/jcs.109.4.713

[54] M. Tanaka , T. Kikuchi , H. Uno , K. Okita , T. Kitanishi‐Yumura , S. Yumura , Sci. Rep. 2017, 7, 1297. 29021607 10.1038/s41598-017-13438-5 PMC5636814

[55] E. Potthoff , D. Franco , V. D'Alessandro , C. Starck , V. Falk , T. Zambelli , J. A. Vorholt , D. Poulikakos , A. Ferrari , Nano Lett. 2014, 14, 1069. 24428164 10.1021/nl4047398

[56] E. Tkachenko , E. Gutierrez , S. K. Saikin , P. Fogelstrand , C. Kim , A. Groisman , M. H. Ginsberg , Biol. Open 2013, 2, 1007. 24167710 10.1242/bio.20134622 PMC3798183

[57] F. Milde , D. Franco , A. Ferrari , V. Kurtcuoglu , D. Poulikakos , P. Koumoutsakos , Integr. Biol. 2012, 4, 1437. 10.1039/c2ib20113e 23047374

[58] J. P. Butler , I. M. Tolic‐Norrelykke , B. Fabry , J. J. Fredberg , Am. J. Physiol. Cell Physiol. 2002, 282, C595. 11832345 10.1152/ajpcell.00270.2001

[59] S. V. Costes , D. Daelemans , E. H. Cho , Z. Dobbin , G. Pavlakis , S. Lockett , Biophys. J. 2004, 86, 3993. 15189895 10.1529/biophysj.103.038422 PMC1304300

[60] P. Biscari , M. C. Calderer , E. M. Terentjev , Phys. Rev. E: Stat., Nonlinear, Soft Matter Phys. 2007, 75, 05170. 10.1103/PhysRevE.75.051707 17677084

[61] A. K. Bhattacharjee , G. I. Menon , R. Adhikari , Phys. Rev. E: Stat., Nonlinear, Soft Matter Phys. 2008, 78, 02670. 10.1103/PhysRevE.78.026707 18850973

