# Supplementary material for: Bistability of Dielectrically Anisotropic Nematic Crystals and the Adaptation of Endothelial Collectives to Stress Fields
Source: Adv Sci (Weinh). 2022 Mar 28;9(16):2102148. doi: 10.1002/advs.202102148 (PMC9165505; doi:10.1002/advs.202102148)
Supplement: Supplementary file 1 — Supporting Information [file ADVS-9-2102148-s001.pdf]

ADVANCED SCIENCE
Open Access

Supporting Information

for *Adv. Sci.*, DOI 10.1002/advs.202102148

Bistability of Dielectrically Anisotropic Nematic Crystals and the Adaptation of Endothelial Collectives to Stress Fields

Georgios Stefopoulos, Tobias Lendenmann, Thomas M. Schutzius, Costanza Giampietro, Tamal Roy, Nafsika Chala, Fabio Giavazzi, Roberto Cerbino, Dimos Poulikakos* and Aldo Ferrari**

Supporting Information

Bistability of dielectrically anisotropic nematic crystals and the adaptation of endothelial collectives to stress fields

Georgios Stefopoulos, Tobias Lendenmann, Thomas M. Schutzius*, Costanza Giampietro, Tamal Roy, Nafsika Chala, Fabio Giavazzi, Roberto Cerbino, Dimos Poulikakos*, Aldo Ferrari*

S1. Modeling Nematic Liquid Crystals

For the polar nematic liquid crystal simulations, the free energy is defined as

$F = \int d^3r(F_I + F_{II} + F_{III})$, where **r** is the position and the three terms are defined as

$$F_I = \frac{A}{2} trQ^2 + \frac{B}{3} trQ^3 + \frac{C}{4}(trQ^2)^2 + \frac{D}{6}(trQ^3)^2, \quad \text{(S1)}$$

and is obtained from a local expansion of the rotationally invariant powers of the order parameter where *A* (nematic vs. isotropic), *B* (rod vs. disk molecules), *C* (greater than zero; ensures that the free energy density functional is bounded from below; [60]), and *D* (uniaxial vs biaxial) are nematic material parameters and

$$F_{II} = \frac{R_1}{2} \partial_\alpha Q_{\beta\gamma} \partial_\alpha Q_{\beta\gamma}, \quad \text{(S2)}$$

(Einstein notation) which is non-local and is obtained rotationally invariant combinations of gradients of the order parameter where R_1 is an elastic constant and

$$F_{III} = -\Delta\varepsilon Q_{\alpha\beta} E_\alpha E_\beta, \quad \text{(S3)}$$

which is due to the effect of the electric field.

For the simulations, we worked with a [N_x, N_y, N_z] = [64, 64, 1] grid; the constants *A*, *B*, *C*, *D*, and R_1, were set to ±1, -1, 2.67, 0, and 1, respectively. All other values that were used are given in the figures. The kinetic coefficient, Γ, was set to 1.0. To calculate the global free energy, we computed the local value at each point and summed at every time point until the final time t_{end}, which was set to 500. The spatial steps, *dx*, *dy*, *dz* were set to 1.0, 1.0, and 1.0, respectively, and the time step *dt* was 0.1. For this model, we made one of the above constant

B inversely related to the frequency of the electric field. When $\omega = \omega_0$, B=1, and when $\omega \neq \omega_0$, B=-1.

S2. Analogy Between the *in silico* Model and *in vitro* Experiments

The electric (|**E**|) and wall shear stress (|**τ**|) field magnitudes control the shape of the particles (phenomenological parameter, B, [61]) and cells aspect ratio, respectively. For the case where |**E**|=0 or |**τ**|=0, this leads to disk-like particles and cells with low aspect ratio, respectively, while non-zero fields lead to rod-like particles or high-aspect ratio cells, respectively. When the fields are non-zero, their direction also controls the particles and cell polarization (i.e. the PCP), which is always in the direction opposite of the imposed fields. In the case of the particles, we assume they are dual-frequency nematic, and the frequency of the external field determines whether they polarize along the long or short axis of the particle. [35] For the cells, we found experimentally that the magnitude of the shear stress determines whether they polarize (i.e. displace their Golgi apparatus relative to the cell nucleus) along their long or short axis. Therefore, the WSS in the cells and the electric field frequency are related from a pure analogical point of view where the systems show order-disorder transitions based on the magnitudes of the control parameters. The endothelial analogue of the dielectric anisotropy arises from different mechanosensitive pathways, which are responsible for disruption of cell-to-cell junctions and affect monolayer integrity.

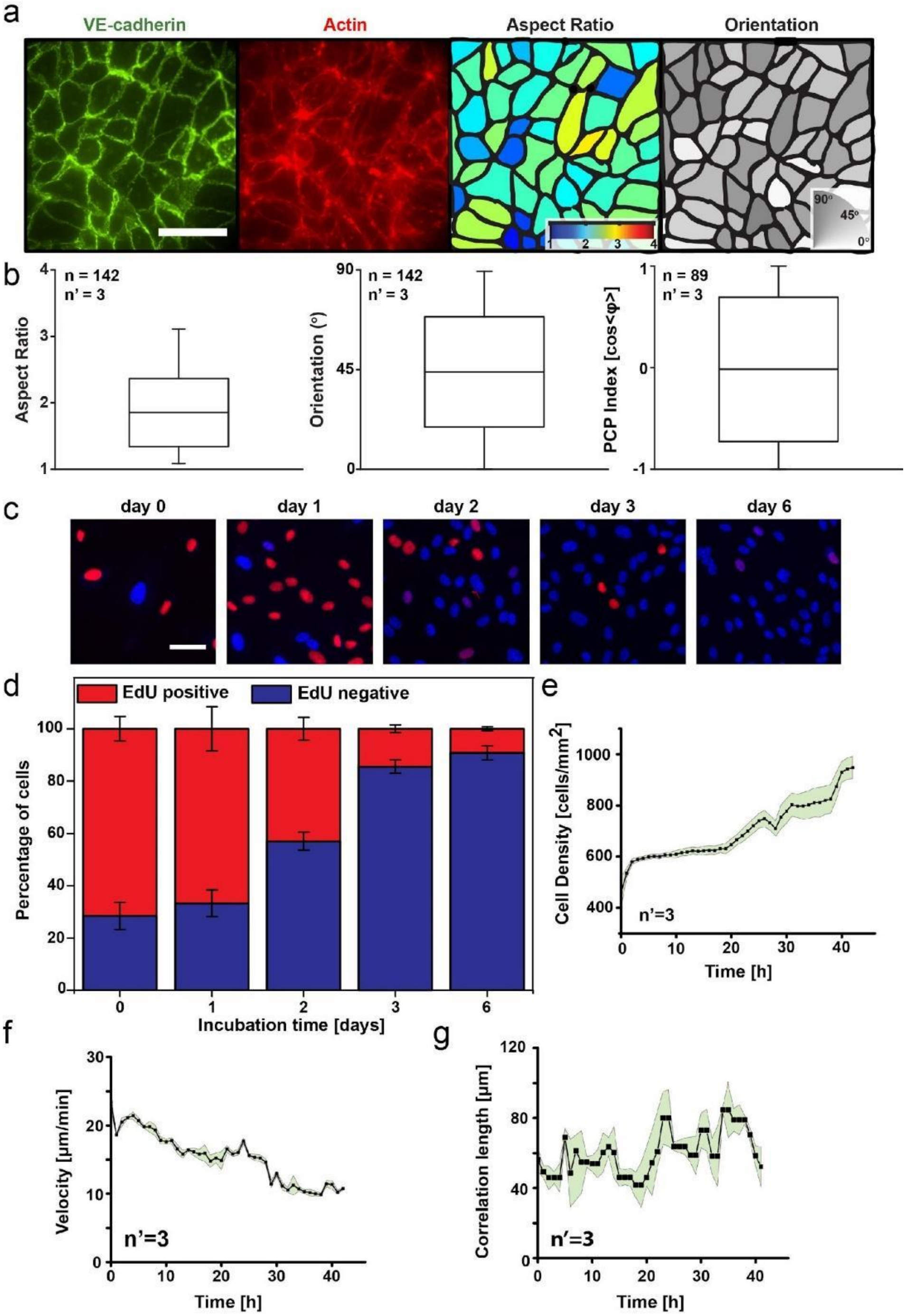

Figure S1. Generation of mature endothelial monolayers. (**a**) Isotropic state obtained in static conditions. Fluorescent images of VE-cadherin distribution (green) at cell-to-cell junctions, and actin cytoskeleton (red). Scale bar is 50 µm. Cell profiles with color-coded aspect ratio, and cell orientation as encoded by corresponding color scale bars. (**b**) Corresponding boxplots reporting the quantification of cell aspect ratio (left), orientation (middle) and PCP index (right). (**c-d**) Cell proliferation during monolayer maturation. (c) Fluorescent images and (d) quantification of EdU assay. Time evolution of (**e**) cell density, (**f**) velocity, and (**g**) correlation length. The shaded area indicates the standard deviation. The number of independent experiments is indicated as n'.

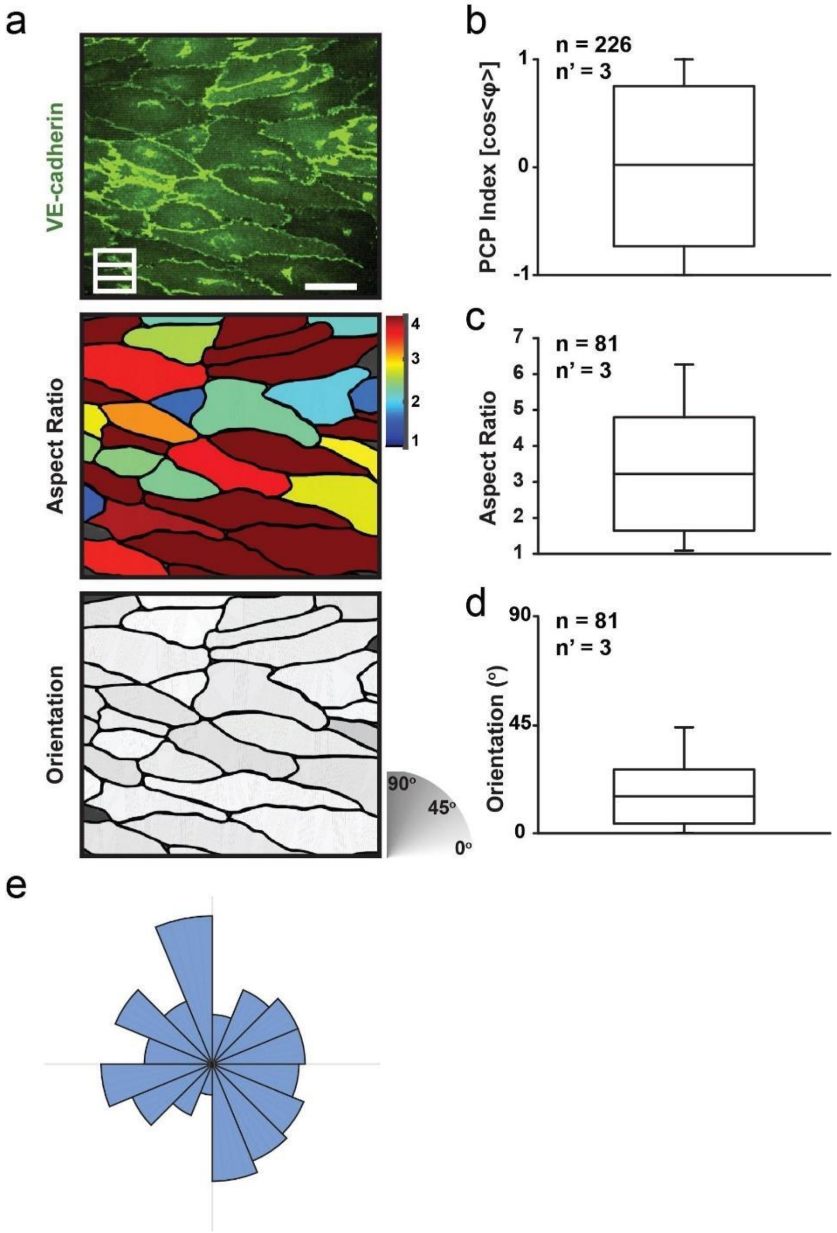

Figure S2. Anisotropic endothelial monolayer generated in static conditions on microstructured gratings. (**a**) Fluorescent images of VE-cadherin distribution (green; top) at cell-to-cell junctions. Scale bar is 50 μm. The orientation of the gratings is reported in the lower left corner. Cell profiles with color-coded aspect ratio (middle), and cell orientation (bottom) as encoded by corresponding color scale bars. Corresponding boxplots reporting the quantification of (**b**) PCP index, (**c**) cell aspect ratio, and (**d**) orientation. (**e**) Radial distribution of PCP (i.e. ϕ as defined in Fig. 1h). The number of analyzed fields of view is reported as n and the number of independent experiments as n'.

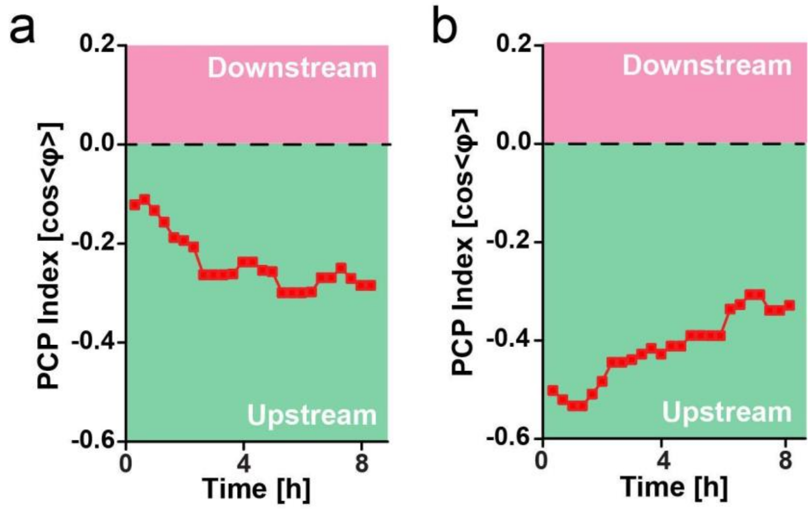

Figure S3. Evolution of planar cell polarity (PCP) upon system perturbation. (**a**) PCP dynamics upon flow intensity increase (WSS values from 1.4 Pa to 8 Pa) or (**b**) decrease (WSS values from 8 Pa to 1.4 Pa).

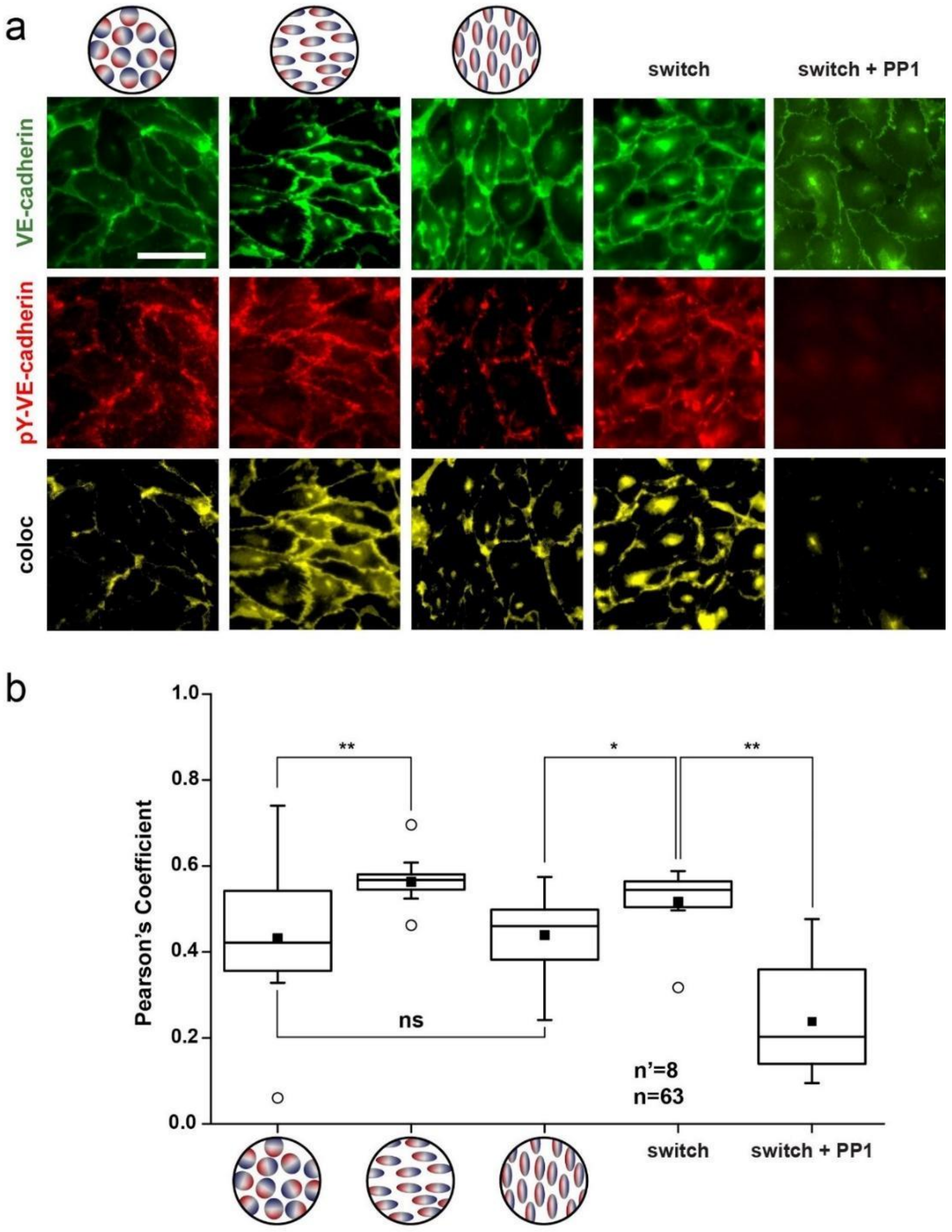

Figure S4. Flow-mediated phosphorylation of VE-cadherin reporting on the dynamic instability of cell-to-cell junctions. (**a**) Fluorescent images depicting the distribution of total VE-cadherin (green, top row), pY658-VE-cadherin (red, middle row) and of their colocalization (yellow, last row). Panels from left to right display endothelia under static conditions (static), exposed to flow yielding WSS of 1,4 Pa (parallel) or 8 Pa (perpendicular), and to system perturbation alone (switch) or in presence of a Src kinase inhibitor (switch + PP1). A corresponding representation of the endothelial monolayer states is displayed in the circular cartoons. The direction of flow direction is indicated by a white arrow. (**b**) Boxplots reporting the measured Pearson's correlation coefficient between VE-cadherin and phosphorylated VE-cadherin (py-VEC) channels for each condition. A corresponding representation of the endothelial monolayer states is displayed in the circular cartoons. The number of analyzed fields of view is reported as n and the number of independent experiments as n'.

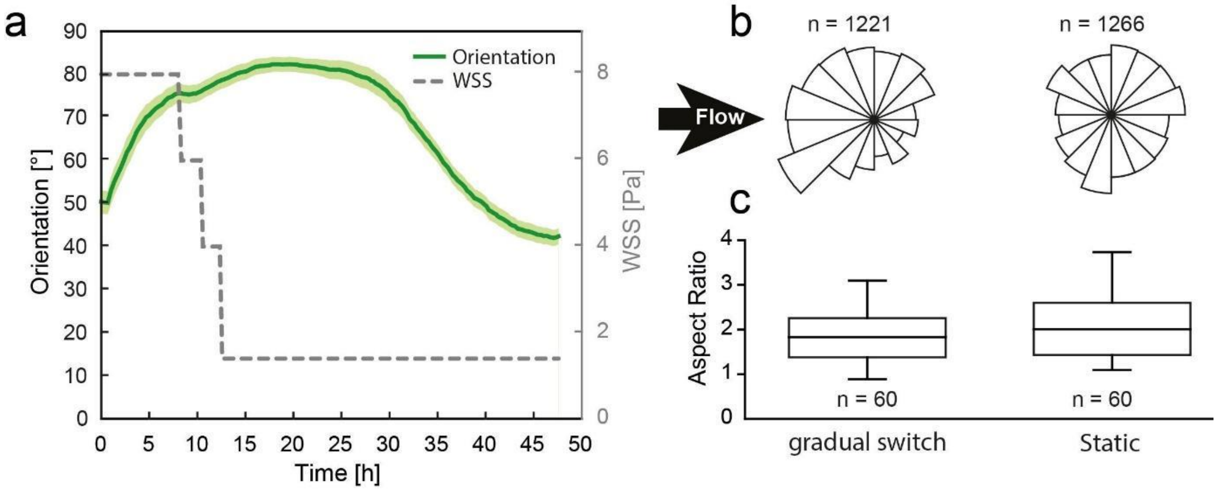

Figure S5. System transitions upon gradual WSS switch. **a**) Evolution of cell orientation upon onset of unidirectional flow (Time = 0 to 8 h) generating high (i.e. 8 Pa) wall shear stress (WSS) followed by a gradual switch to 6 (Time = 8 to 10 h), 4 (Time = 10 to 12 h) and 1.4 Pa (Time = 12 to 48 h). The corresponding WSS value is indicated by a dashed gray line. Endpoint radial distribution of **b**) PCP (i.e. ϕ as defined in Fig. 1h) and **c**) aspect ratio as compared to a static control.

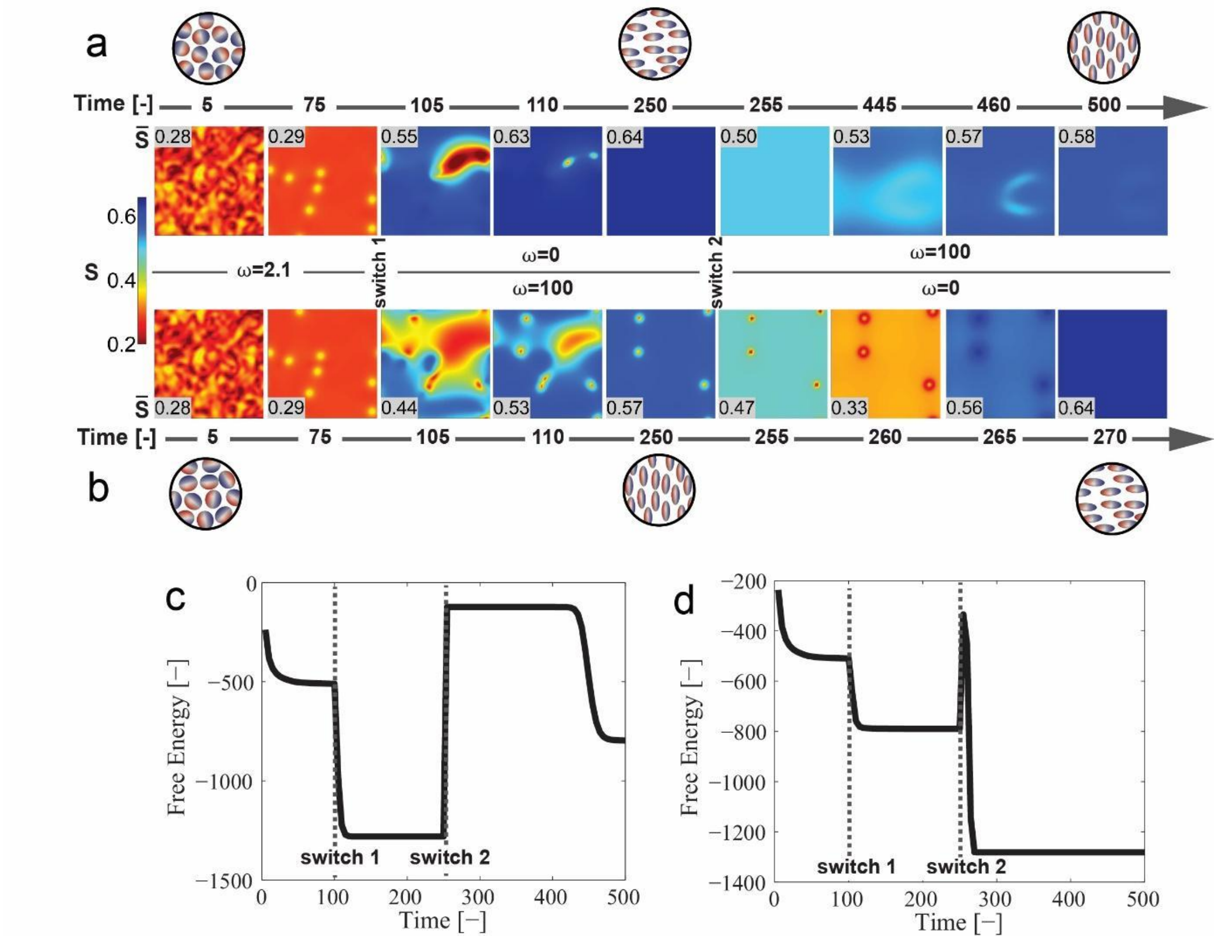

Figure S6. Robustness of the numerical scheme for the system transitions with extreme values of ω where ω_L=0 s^{-1} and ω_H=100 s^{-1}. Here A=-1, B=1 for Time<100, B=-1 for Time≥100, C=2.67, D=0, $\varepsilon_{\parallel,0}= 0.5$, $\varepsilon_{\parallel,\infty}= 0.01$, $\varepsilon_{\perp}= 0.2$ and $\tau= 0.6\ s$. (**a**) Heat maps of the transient nematic order parameter, S, for switching of ω from ω_0 to ω_L ($\Delta\varepsilon > 0$) and subsequently from ω_L to ω_H ($\Delta\varepsilon < 0$). (**b**) Corresponding heat maps for switching of ω from ω_0 to ω_H ($\Delta\varepsilon < 0$) and subsequently from ω_H to ω_L ($\Delta\varepsilon > 0$). The mean value $\underline{S}$ is shown in the inset. Both (a) and (b) are initiated with the same random configuration. At time = 100 and 250 the frequency changes are triggered (switch 1 and switch 2, respectively). A corresponding representation of the initial (left), intermediate (middle) and final (right) states is displayed in the circular cartoons. The magnitude of S is indicated by the color scale bar. (**c**) Free energy profile during the system transition depicted in (a). (**d**) Corresponding free energy profile during the transition depicted in (b).

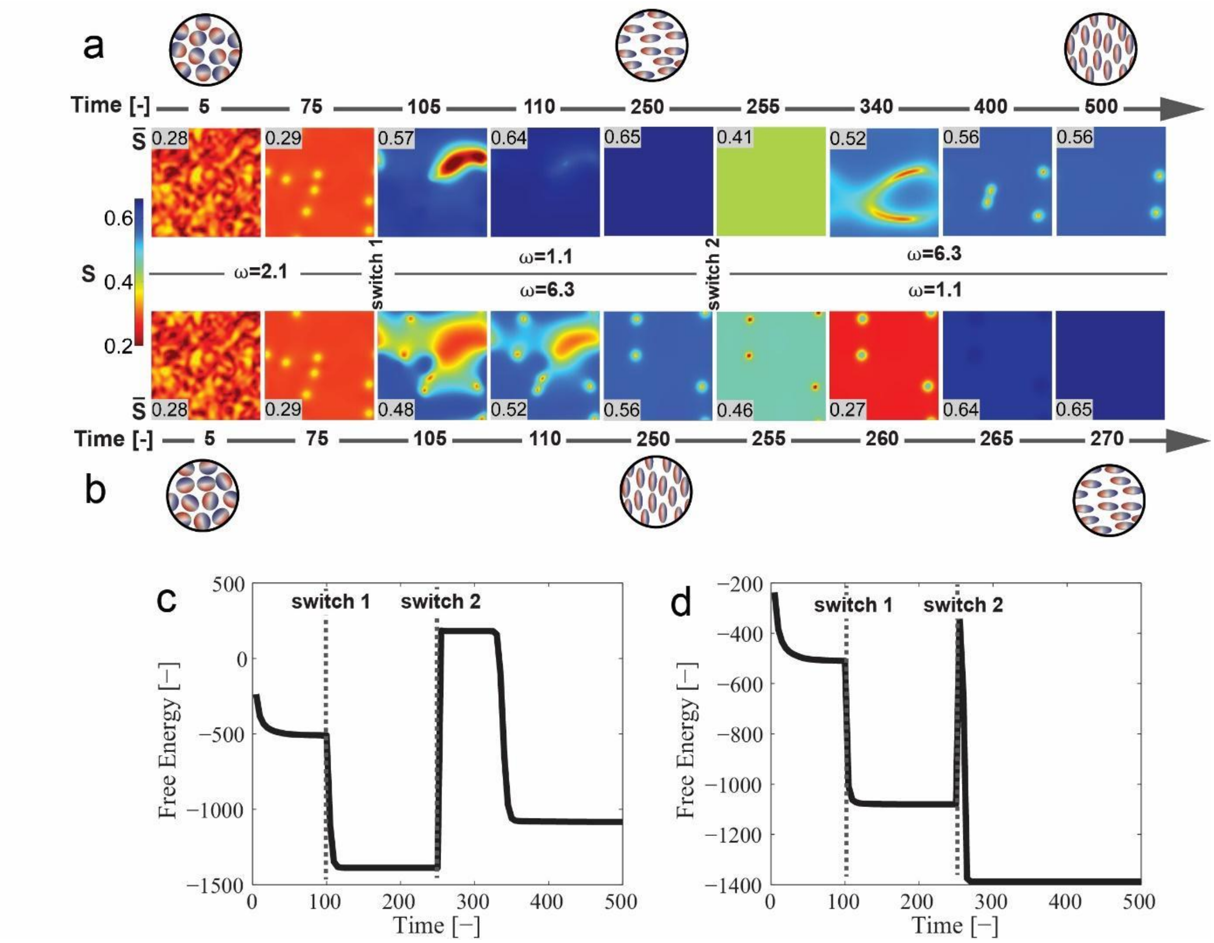

Figure S7. Robustness of the numerical scheme for the system transitions with E_x=1.5 Vm^{-1}. Here A=-1, B=1 for Time<100, B=-1 for Time≥100, C=2.67, D=0, $\varepsilon_{\parallel,0} = 0.5$, $\varepsilon_{\parallel,\infty} = 0.01$, $\varepsilon_{\perp} = 0.2$ and $\tau = 0.6\ s$. (**a**) Heat maps of the transient nematic order parameter, S, for switching of ω from ω_0 to ω_L($\Delta\varepsilon > 0$) and subsequently from ω_L to ω_H ($\Delta\varepsilon < 0$). (**b**) Corresponding heat maps for switching of ω from ω_0 to ω_H ($\Delta\varepsilon < 0$) and subsequently from ω_H to ω_L ($\Delta\varepsilon > 0$). The mean value $\underline{S}$ is shown in the inset. Both (a) and (b) are initiated with the same random configuration. At time = 100 and 250 the frequency changes are triggered (switch 1 and switch 2, respectively). A corresponding representation of the initial (left), intermediate (middle) and final (right) states is displayed in the circular cartoons. The magnitude of S is indicated by the color scale bar. (**c**) Free energy profile during the system transition depicted in (a). (**d**) Corresponding free energy profile during the transition depicted in (b).

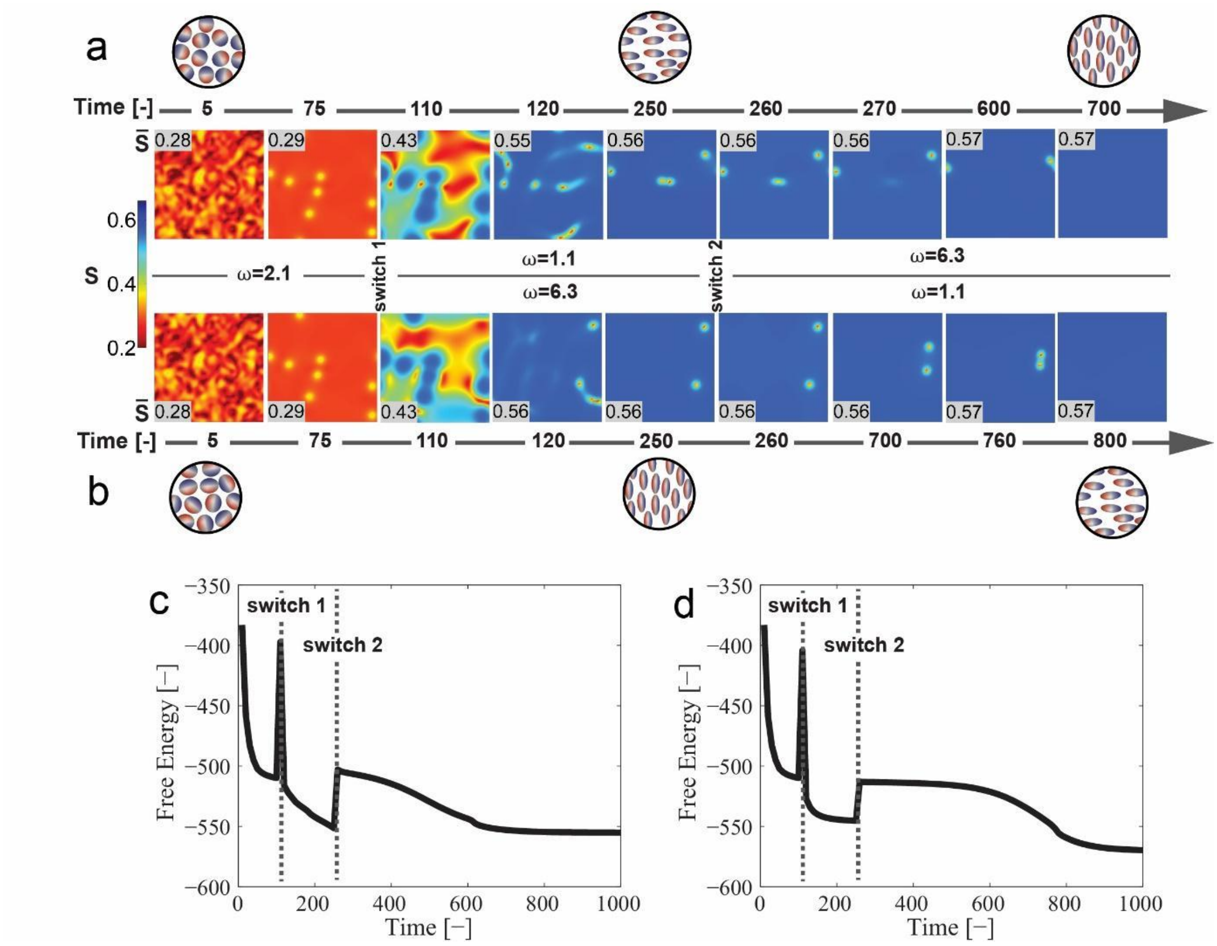

Figure S8. Robustness of the numerical scheme for the system transitions with E_x=0.3 Vm^{-1}. Here A=-1, B=1 for Time<100, B=-1 for Time≥100, C=2.67, D=0, $\varepsilon_{\parallel,0} = 0.5$, $\varepsilon_{\parallel,\infty} = 0.01$, $\varepsilon_{\perp} = 0.2$ and $\tau = 0.6\ s$. (**a**) Heat maps of the transient nematic order parameter, S, for switching of ω from ω_0 to $\omega_L(\Delta\varepsilon > 0)$ and subsequently from ω_L to ω_H $(\Delta\varepsilon < 0)$. (**b**) Corresponding heat maps for switching of ω from ω_0 to ω_H $(\Delta\varepsilon < 0)$ and subsequently from ω_H to ω_L $(\Delta\varepsilon > 0)$. The mean value $\underline{S}$ is shown in the inset. Both (a) and (b) are initiated with the same random configuration. At time = 100 and 250 the frequency changes are triggered (switch 1 and switch 2, respectively). A corresponding representation of the initial (left), intermediate (middle) and final (right) states is displayed in the circular cartoons. The magnitude of S is indicated by the color scale bar. (**c**) Free energy profile during the system transition depicted in (a). (**d**) Corresponding free energy profile during the transition depicted in (b).

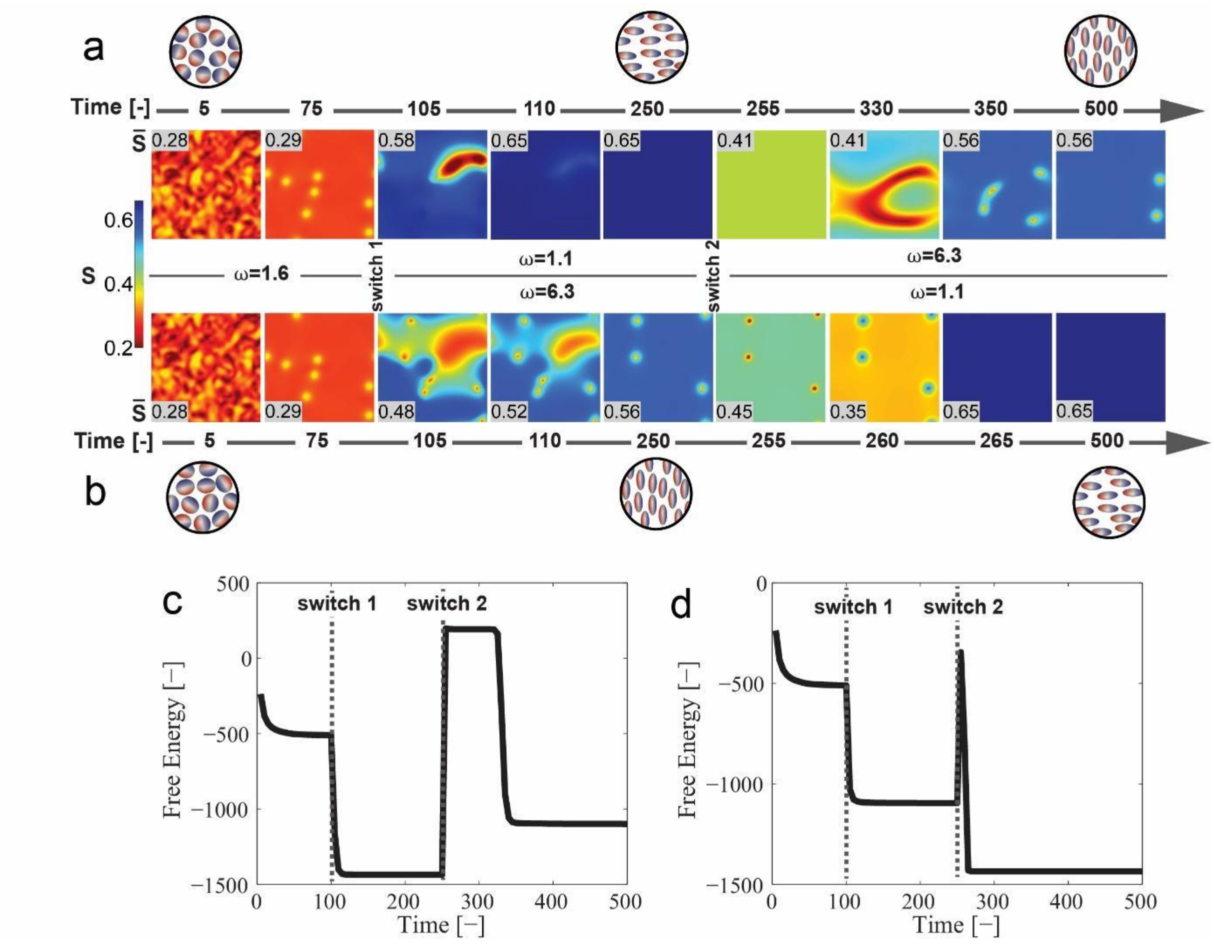

Figure S9. Robustness of the numerical scheme for the system transitions with $|\Delta\varepsilon| = 0.36$, where $\varepsilon_{\parallel,0} = 4.75$, $\varepsilon_{\parallel,\infty} = 0.01$, $\varepsilon_{\perp} = 0.4$ and $\tau = 2.1$. Here *A*=-1, *B*=1 for Time<100, *B*=-1 for Time≥100, *C*=2.67, *D*=0. (**a**) Heat maps of the transient nematic order parameter, S, for switching of ω from ω_0 to ω_L ($\Delta\varepsilon = 0.36$) and subsequently from ω_L to ω_H ($\Delta\varepsilon = -0.36$). (**b**) Corresponding heat maps for switching of ω from ω_0 to ω_H ($\Delta\varepsilon = -0.36$) and subsequently from ω_H to ω_L ($\Delta\varepsilon = 0.36$). The mean value $\underline{S}$ is shown in the inset. Both (a) and (b) are initiated with the same random configuration. At time = 100 and 250 the frequency changes are triggered (switch 1 and switch 2, respectively). A corresponding representation of the initial (left), intermediate (middle) and final (right) states is displayed in the circular cartoons. The magnitude of S is indicated by the color scale bar. (**c**) Free energy profile during the system transition depicted in (a). (**d**) Corresponding free energy profile during the transition depicted in (b).

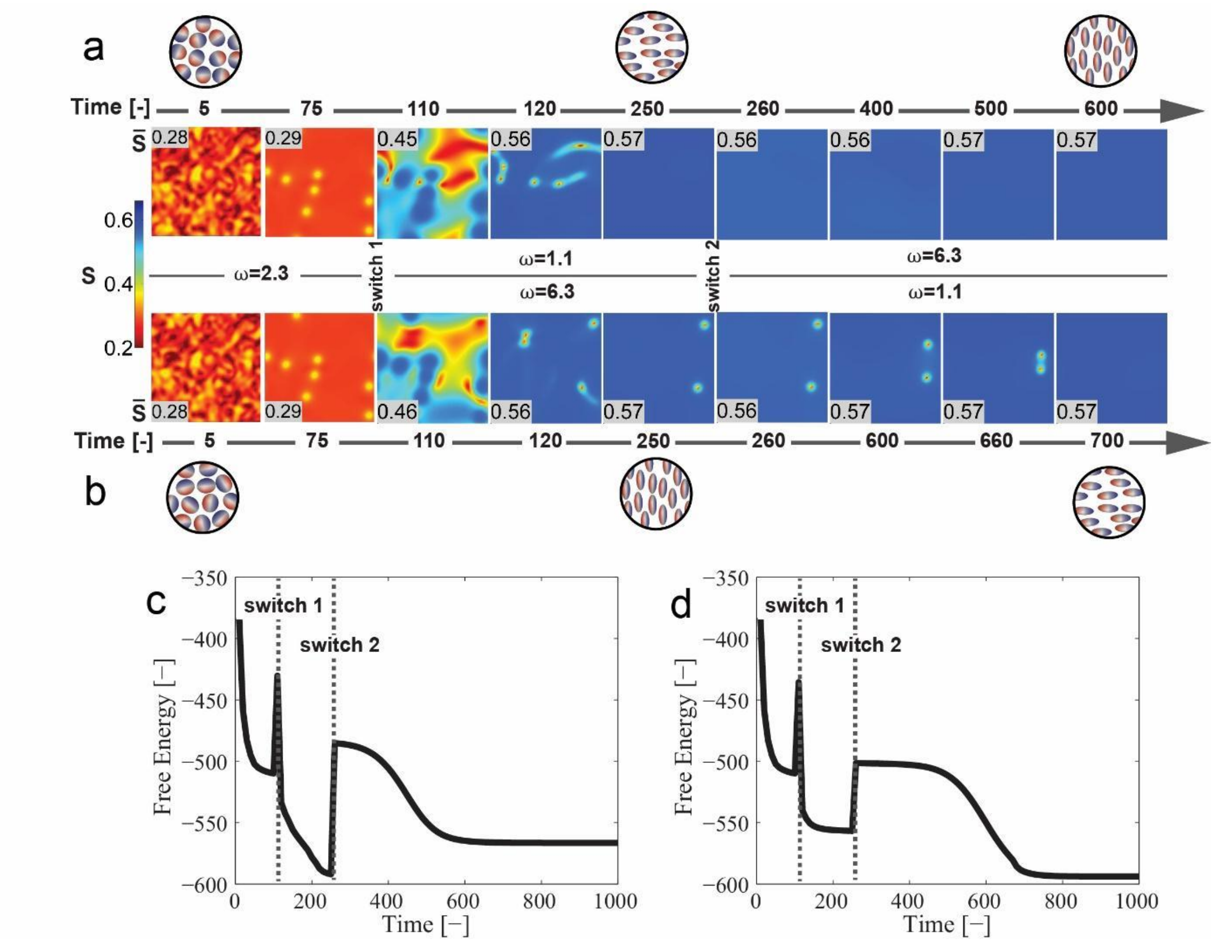

Figure S10. Robustness of the numerical scheme for the system transitions with $|\Delta\varepsilon| = 0.02$, where $\varepsilon_{\parallel,0} = 0.08$, $\varepsilon_{\parallel,\infty} = 0.01$, $\varepsilon_{\perp} = 0.04$ and $\tau = 0.5$. Here *A*=-1, *B*=1 for Time<100, *B*=-1 for Time≥100, *C*=2.67, *D*=0. (**a**) Heat maps of the transient nematic order parameter, S, for switching of ω from ω_0 to ω_L ($\Delta\varepsilon = 0.02$) and subsequently from ω_L to ω_H ($\Delta\varepsilon = -0.02$). (**b**) Corresponding heat maps for switching of ω from ω_0 to ω_H ($\Delta\varepsilon = -0.02$) and subsequently from ω_H to ω_L ($\Delta\varepsilon = 0.02$). The mean value $\underline{S}$ is shown in the inset. Both (a) and (b) are initiated with the same random configuration. At time = 100 and 250 the frequency changes are triggered (switch 1 and switch 2, respectively). A corresponding representation of the initial (left), intermediate (middle) and final (right) states is displayed in the circular cartoons. The magnitude of S is indicated by the color scale bar. (**c**) Free energy profile during the system transition depicted in (a). (**d**) Corresponding free energy profile during the transition depicted in (b).

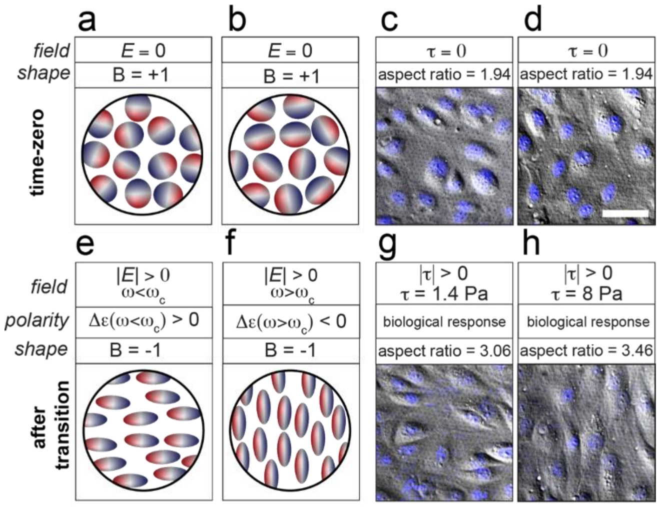

Figure S11. Analogy between *in silico* model of dielectrically anisotropic liquid crystals and i*n vitro* experiments of the confluent monolayer of young and healthy endothelial cells. Initial conditions for the (**a**)-(**b**) *in silico* model and (**c**)-(**d**) *in vitro* experiments. Conditions that induce the transition for the (**e**)-(**f**) *in silico* model and (**g**)-(**h**) *in vitro* experiments. Scale bar: (**d**) 50µm.

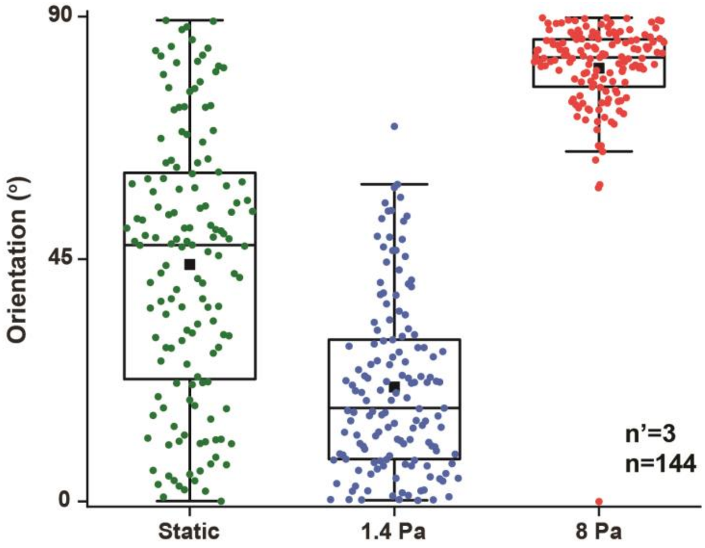

Figure S12. The full dataset of three independent experiments of young and healthy ECs alignment to flow is reported. The number of analysed cells is reported as n and the number of independent experiments as n'. In box plots the population means are reported as a square inside the boxplot, the population medians are reported as a horizontal line inside the boxplot, the box extends from the 25th to the 75th percentiles and whiskers report the 1.5 x IQR (inter-quantile range).

Table SI: *In vitro* and *in silico* parameter correspondence. Here WSS is the *in vitro* wall shear stress; E, ω and B are in silico electric field amplitude, frequency and nematic shape parameter, respectively.

In vitro		*In silico*					
WSS [Pa]	0	E	[1 0 0]	ω	2.16	B	1
	1.4		[1 0 0]		1.1		-1
	8		[1 0 0]		6.3		-1
